# Melt-induced buoyancy may explain the elevated rift-rapid sag paradox during breakup of continental plates

**DOI:** 10.1038/s41598-018-27981-2

**Published:** 2018-07-03

**Authors:** David G. Quirk, Lars H. Rüpke

**Affiliations:** 1 Manx Geological Survey/University of Manchester, Gammel Mønt 31, 1117 Copenhagen K, Denmark; 2 GEOMAR Helmholtz Centre for Ocean Research Kiel, Wischhofstraße 1-3, 24148 Kiel, Germany

## Abstract

The division of the earth’s surface into continents and oceans is a consequence of plate tectonics but a geological paradox exists at continent-ocean boundaries. Continental plate is thicker and lighter than oceanic plate, floating higher on the mantle asthenosphere, but it can rift apart by thinning and heating to form new oceans. In theory, continental plate subsides in proportion to the amount it is thinned and subsequently by the rate it cools down. However, seismic and borehole data from continental margins like the Atlantic show that the upper surface of many plates remains close to sea-level during rifting, inconsistent with its thickness, and subsides after breakup more rapidly than cooling predicts. Here we use numerical models to investigate the origin and nature of this puzzling behaviour with data from the Kwanza Basin, offshore Angola. We explore an idea where the continental plate is made increasingly buoyant during rifting by melt produced and trapped in the asthenosphere. Using finite element simulation, we demonstrate that partially molten asthenosphere combined with other mantle processes can counteract the subsidence effect of thinning plate, keeping it elevated by 2-3 km until breakup. Rapid subsidence occurs after breakup when melt is lost to the embryonic ocean ridge.

## Introduction

The aim of this paper is to test the geological causes of problematic subsidence patterns at the margins of ocean basins. The established rift paradigm explains the formation of ocean basins by stretching, thinning and breakup of continental plate or lithosphere. As the crust thins, it subsides due to decreasing buoyancy while the asthenosphere rises to fill the extensional space created beneath the necking lithosphere. When rifting ceases, the crust then sinks further as it and the underlying mantle cool, a model elegantly enumerated by McKenzie^1^. There is however a problem with subsidence patterns on rifted margins as they often do not fit the predicted response of the standard McKenzie stretching-cooling model, with too little syn-rift strata (deposited when extensional faults were active) and too much “sag” strata, deposited soon after faults in the crust had ceased to move^2^. A related but equally puzzling issue has become apparent in palaeo-water depths, where the widespread occurrence of continental and shallow water (<200 m) sediments, erosion surfaces and subaerial volcanics are recorded at the time of breakup^3–5^, indicating that the surface of the plate is not only relatively flat but also anomalously elevated, up to 2600 m higher than normal oceanic crust, in conflict with common physical assumptions for crust and mantle^1^. Regions with unexplained elevation at the time of breakup include Brazil and its conjugate margin Gabon-Angola-Namibia^6^, the Gulf of Mexico^7^, the North Atlantic^5^, Middle East-Arabian Gulf^8^ and western Australia^3^. Subsequently, when sea-floor spreading starts, rapid subsidence occurs to oceanic depths within 3–20 million years^7^, much faster than can be explained by post-rift thermal contraction. One way of addressing such plate-scale problems is to build numerical models^9–12^, where different geodynamic possibilities can be validated or rejected. However, it seems only one numerical model directly addresses the elevated breakup issue (ref.^3^) which shows that dynamic uplift caused by the fountaining effect of rising asthenosphere is an order of magnitude too small. Furthermore, we do not know of any models designed specifically to solve the issue of rapid sag after breakup, although explanations have been proposed such as continued extension below the crust^7,13,14^. We address this shortfall here, by investigating mechanisms which might keep the plate buoyant as it thins and subsequently cause it to subside quickly.

Compared to the crust, the mantle is thick and therefore has the potential to significantly influence the buoyancy of the plate. One plausible scenario explaining the anomalous subsidence patterns is that the mantle gets lighter as rifting proceeds^15,16^. Gravity measurements indicate that significant density variations exist in the mantle, greater than can be explained by thermal or chemical variations^17^: as high as 3400 kg.m^−3^ around subduction zones or as low as 3100 kg.m^−3^ in modern rifts^18^. Anomalously low mantle densities correlate with high surface elevations in continental rifts such as the Ethiopian plateau (2500 m above sea-level, ASL) and Kenya dome (1900m ASL) where upwelling and partially molten asthenosphere has partially displaced the original mantle lithosphere^18–20^ and thickened it with igneous intrusions^21–23^. Changes to lighter mineral phases as dry mantle rises during rifting have already been modeled^15,16^ but we will show later that, by themselves, these are insufficient to keep the crust elevated by more than 1000 m. Geophysical interpretations of the Transantarctic Mountains have suggested a link between elevation and the thermal effect of rift-related melting^24^ although the reduction in density caused by the melt itself has not yet been considered.

There is typically a 3–5 km difference in the height between continental rifts and oceanic rifts (spreading centres) related to a difference in their buoyancy (isostatic equilibrium). Although it becomes thinner as it stretches, the plate in continental rifts is composed of granitic crust underlain by thick mantle lithosphere. This mantle lithosphere neither melts nor circulates easily and is therefore chemically and rheologically different to the mantle asthenosphere beneath the plate. It is also colder than the asthenosphere, conducting rather than convecting heat and is relatively impermeable to melt^25^, magma transported upwards along fractures and faults rather than by melt flowing through a pore network^19,20,23^. In contrast, along the rift axis of most mid-ocean ridges the mantle lithosphere is absent: the ridge connected instead to shallow, upwelling asthenosphere where the melt responsible for oceanic crust is generated^26^, which flows upwards through pores^11,27^.

We now hypothesize that the relative impermeability of mantle lithosphere to melt may be the root cause of additional elevation. Thus, as continental plate thins during rifting, the asthenosphere wells up and pressure is reduced causing it to partially melt in proportion to the height the asthenosphere rises^17,25,26^. Melt is lower density than the parent asthenosphere^28^ and will tend to migrate upwards through the asthenosphere by compaction once a percolation threshold defined by melt-filled porosity is crossed^29^. However, the overlying mantle lithosphere will act as a barrier, impeding its upward flow^12,19,23,25^. This opens the possibility that significant amounts of melt are pooled in the asthenosphere^30,31^ making it buoyant^17^ so that the overlying lithosphere rises^18,19,22,23^. When the rifting plate breaks, the melt is then lost to the embryonic ocean ridge as seafloor spreading starts.

The research reported here concerns rifted margins associated with normal opening rates, using a specific example from the central South Atlantic, generally regarded as non-volcanic. We do not discuss rifted margins associated with very slow separation, where melting is suppressed and mantle can be exhumed^10^.

## Geological Background

**Figure 1:**
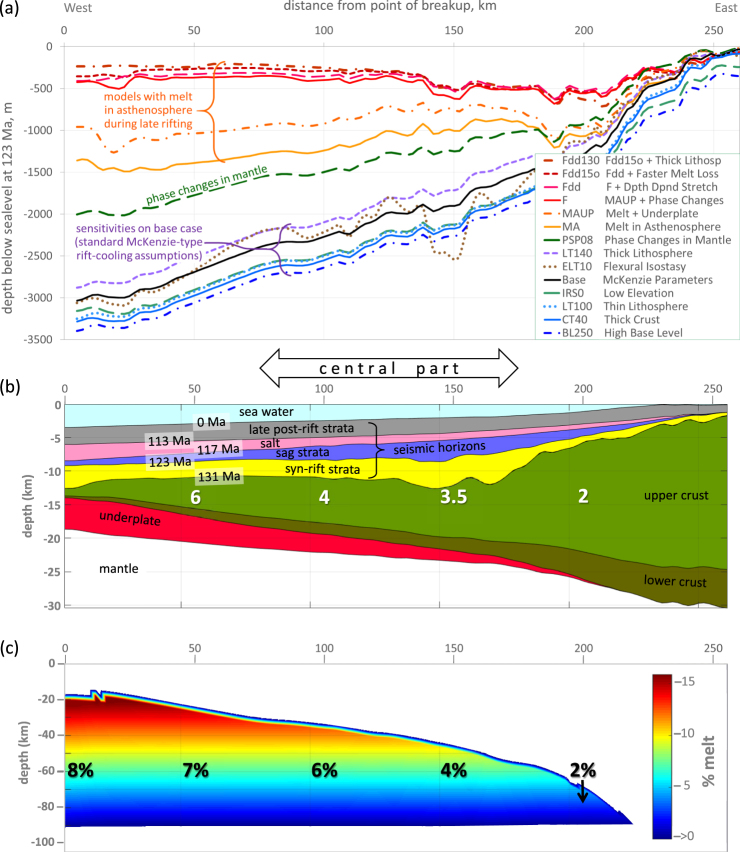
Cross-sections from 2D thermo-tectonostratigraphic models of a non-volcanic rifted margin (Kwanza Basin, offshore Angola). (**a**) Depth of syn-rift sediment surface at time of breakup, 123 Ma, for models where the stratigraphic match is good-perfect (Table 1). (**b**) Geological section showing the results of model Fdd15o which perfectly matches the input sediment thicknesses after incorporating melt, underplating, phase changes and minor depth dependent stretching. White numbers – stretching factor for crust (original thickness divided by present thickness). Syn-rift strata comprise continental sediments, sag strata comprise shallow water (<200 m) carbonates and lacustrine mudstones, late post-rift strata comprise marine sediments. (**c**) Melt structure in model Fdd15o at time of breakup, color indicating the amount of melt at each point in the asthenosphere. Average melt percentages are indicated (black font).

The aim of our models is to test different concepts which might explain the elevation-subsidence paradox associated with continental breakup, including changes in density in the asthenosphere (Fig. 1 and Table 1). There are many uncertainties in interpretations of rifted margins but there is little argument that water depths and subsidence rates around the time of breakup do not fit with the standard McKenzie stretching-cooling model^1^ which has been used in basin analysis for the last 40 years. We choose to focus on the central South Atlantic as this contains one of the world’s best studied rifted margins since the discovery of massive oil accumulations in strata associated with continental breakup. Over the last 12 years, the region has been blanketed by high quality seismic data and penetrated by hundreds of boreholes in the search for new hydrocarbon resources within shallow water lacustrine carbonates belonging to the “sag” interval. The sag directly underlies thick salt and both are Aptian in age, c.123-113 million years ago (Ma)^6,32,33^ based on palaeontological data. The salt is overlain by marine carbonates and shales which record both deepening and tilting of the seabed towards the ocean as the basin subsided. In contrast, both the sag and the salt contain unusually uniform strata, comprising individual sediment cycles which can be correlated for hundreds of kilometres^6,32,34^, both across the basin and along it. These strata were deposited in a shallow water, saline lake which shows palaeontological evidence for connection to marine waters^33,35^. The base of the sag is marked by an erosional surface at the top of continental rift sediments dated as 131-c.123 Ma^33^, constrained by igneous isotopic ages^32,36^. These syn-rift strata have been rotated and extended by numerous syn-sedimentary normal faults^6^. The erosional surface or unconformity appears to represent a period of subaerial exposure^4,33^.

**Table 1:** Summary of different models of the formation of a non-volcanic rifted margin (Fig. 1) compared to a base case using standard McKenzie-type assumptions^1^.

Model		Stratigraphic match	Difference in water depth, m, relative to base case at 10 km from breakup point
Base Case	35 km crust, 120 km lithosp, initial rift surface 500 m ASL, zero elastic thickness, no melt, no intrusions, homogeneous extension	good	
UFB	Underfilled basin (low syn-rift sedimentation rate of 0.1 mm.yr^−1^)	very poor	+1300
MntSerp	Same as MntlEx with maximum amount of serpentinization	very poor	+625
MntlEx	Crust thinned to <1 km between 80–50 km of breakup point, 123 Ma	very poor	+550
BL250	Continental sediment preserved (high syn-rift lake level + 250 m ASL)	perfect	+375
CT40	Crust 40 km thick	perfect	+275
LT100	Lithosphere 100 km thick	perfect	+225
bR144	Earlier start of rift (144 Ma instead of 131 Ma)	perfect	+175
IRS0	Initial rift surface at 0 m above sea-level at 131 Ma	good	+150
tR113	Later start of rift (113 Ma instead of 123 Ma)	moderate	+100
bR136	Earlier start of rift (136 Ma instead of 131 Ma)	perfect	+75
tR117	Later start of rift (117 Ma instead of 123 Ma)	good	+50
ELT10	Elastic thickness of 10 km, necking depth 15 km	good	+25
S2My	Salt deposition period halved (2 My instead of 4 My)	perfect	0
UP	Underplating (total of 1–6 km thick sills intruded 129–123 Ma)	moderate	0
Rs1k	Base of rift 1 km shallower across entire interpetation	good	−25
DD	Depth dependent stretching (mantle thinned 2x more than crust)	moderate	−75
Rs136dd	b rift 1 km shallower, 136 Ma start, depth dependent stretching	good	−100
Rs136	b rift 1 km shallower, 136 Ma start instead of 131 Ma	good	−125
Rd1k	Base of rift 1 km deeper across entire interpretation	moderate	−150
IRS1000	Initial rift surface at +1000 m above sea-level, 131 Ma	moderate	−150
LT140	Lithosphere 140 km thick	good	−150
A3280	Asthenosphere density 3280 kg.m^−3^ instead of 3300 kg.m^−3^	moderate	−350
CT30	Crust 30 km thick	moderate	−500
BL-250	Desiccated basin (syn-rift lake level 250 m BSL)	moderate	−600
BL-1k	Desiccated basin (lake level 1000 m below sea-level at 118 Ma)	poor	−700
PSP08	Phase changes in mantle (R123 peridotite, ref.^16^)	good	−1000
MA	Melt in astheno (max 8% avg 90–20 km then 10% per My melt loss)	good	−1600
MAUP	Melt in astheno + Underplating (≡25% of melt in asthenosphere)	good	−2000
Fnoup	As Fdd15o but with no underplate	good	−2475
F	Melt in astheno + underplating + phase changes in mantle	moderate	−2550
FddR136	As Model Fdd15o but with earlier start of rift (136 Ma not 131 Ma)	good	−2575
Fdd	As Model F with minor depth depnd stretching (mantle ± 25% crust)	perfect	−2600
Fdd15o	As Model Fdd but with 15% per My melt loss after breakup	perfect	−2650
Fdd95k	As Model Fdd15o but with melt starting 5 km deeper at 95 km	perfect	−2700
Fdd1200	As Model Fdd15o but with astheno starting at 1200 °C not 1250 °C	perfect	−2725
Fdd130	As Model Fdd15o but with lithosphere 130 km thick	good	−2775

ASL, above sea-level; BSL, below sea-level.

The sag and salt are surprisingly thick, recording 2->6 km deposited in 10 million years and yet the sediment surface apparently remained flat, close to the surface of the lake, sedimentation rates matching subsidence^32^. In basins with conditions less conducive to the growth of carbonates and without the repeated recharge and evaporation needed for salt deposition, the sediment surface would have subsided to oceanic depths during these 10 million years. The sag itself varies in thickness between 500 m and 5000m^37^, confined to the inboard limits of the rift and outboard by an outer high^38^. This outer high has been interpreted to represent a proto-oceanic ridge comprising thickened oceanic crust^6,34^, the top of which was drilled by Statoil in 2014 and reportedly encountered volcanic basalt of early Aptian age. Despite varying in thickness, the sag has a layer cake-like appearance^6,13,38^: the upper layers being of relatively uniform thickness but the older layers pinching out onto the erosional surface at the top of the syn-rift interval (Fig. S3 in Supplementary Information). This geometry records sequential infill of the basin as water levels rose after the period of surface exposure which marks the end of rift-related faulting.

There are still uncertainties about the structure, thicknesses and ages of the strata beneath the salt, as well as differences in opinion on (i) the age of breakup, (ii) the importance of sub-crustal faults and igneous intrusions and (iii) local lake levels versus global sea-level. Nonetheless, this margin is still better constrained than most, both because of its economic importance and because of the thickness of the sag and salt strata. The unexplained thickness of the sag and the puzzling bathymetric implications of the salt in this region have been the subject of numerous papers since 2007 (see Supplementary Information) but comparable subsidence issues are encountered on many rifted margins at the time of breakup. We therefore use a section from the central South Atlantic to model and explore potential solutions.

## Modeling and Results

The complex interactions of the numerous parameters affecting heatflow and subsidence are difficult to quantify individually. Therefore, we turn to an established basin modeling package, TecMod, which uses pure-shear kinematics in a finite-element technique to simultaneously solve the thickness, temperature, density, isostatic, sedimentary and magmatic evolution of the crust and mantle. The employed method uses an iterative algorithm that automatically refines the stretching and sedimentation history until the modeled section matches the input stratigraphy^39^. We have augmented the standard TecMod routines with a simple parameterization of melting and melt extraction, which allows us to test the isostatic consequence of different magmatic scenarios. This parameterization relates the amount of melt held in the asthenosphere to the height it rises during rifting and allows different assumptions on melt retention and extraction to be tested, including the option that some of the melt is intruded at the base of the crust as sills (“underplating”). The technical details are described in Methods Summary and Supplementary Information. We use a representative cross-section from this region, specifically offshore Angola in the Kwanza Basin, where extensive, deep seismic data of remarkable quality have been acquired. The depth-migrated seismic section we choose extends across the entire rifted margin (Fig. S3 in Supplementary Information) and serves as a generic example of a non-volcanic rifted margin. A series of 2D basin models has been built, attempting to match the subsidence history under different assumptions and using alternative interpretations (Table 1).

Even before modeling, some of the basic issues are apparent on the interpreted section (Fig. 1b); for instance, syn-rift and post-rift thicknesses do not increase sequentially towards the ocean as expected with increased thinning towards the point of breakup. Also, the early post-rift section (123–113 Ma) is disproportionally thick and is composed of shallow water carbonates and mudstones (sag strata) and salt rather than deep marine sediments. Also, unlike post-rift strata associated with thermal subsidence, the sag and salt do not extend beyond the limits of the rift basin^38^. Although the timing of breakup in this region can be debated (see Methods Summary), the thickness and water depth issues are typical of many rifted margins^2,3,5,7,40^ and the section serves as a useful generic example to test ideas.

We have investigated numerous different scenarios to test virtually every conceivable model addressing the apparent subsidence and elevation discrepancy associated with the rifting and continental breakup of the central South Atlantic margin. These include the separate and combined effects of:

- basic McKenzie-type assumptions, with stretching of the plate from 131–123 Ma, followed by cooling (Model Base);
- variations in the height above global sea-level of the initial rift surface (e.g. Models IRS0, IRS1000);
- differences in the pre-rift thickness of the crust and mantle lithosphere (e.g. Models CT30, CT40, LT100, LT140, Fdd130);
- alternative temperatures at the base of the lithosphere (e.g. Model Fdd1200);
- different elastic thicknesses and necking depths which affect flexural isostasy (e.g. Model ELT10);
- alternative interpretations of the thickness of the syn-rift interval (e.g. Models Rd1k, Rs1k, Rs136);
- different assumptions on the age rifting starts and on the age that rifting ends (breakup) (e.g. Models bR144, bR136, tR117, tR113);
- different assumptions on the age of the salt (e.g. Model S2My);
- local variations in lake level assuming that the basin was not connected to the sea during different periods when rift, sag and salt strata were being deposited, allowing the basin to become desiccated or over-filled (e.g. Models BL-1k, BL-250, BL250);
- low sedimentation rates, allowing the basin to become under-filled during rifting (e.g. Model UFB);
- variations in the density of the mantle lithosphere and the asthenosphere (e.g. Model A3280);
- mineral phase transitions due to metamorphism in the mantle lithosphere caused by changes in pressure and temperature (e.g. Models PSP08, all those pre-fixed by F);
- depth dependent stretching (e.g. Models DD, Rs136ddD, all those pre-fixed by Fdd);
- mantle exhumation (e.g. Model MntlEx) and serpentinization (e.g. Model MntSerp);
- underplating or intrusion of sills at the base of the crust (e.g. Models UP, MAUP);
- variations in the amounts of melt retained in the asthenosphere as it rises, in the depth that melting starts and in the rates that melt is lost after breakup (e.g. Models Fdd95k, Fdd15o).

**Figure 2:**
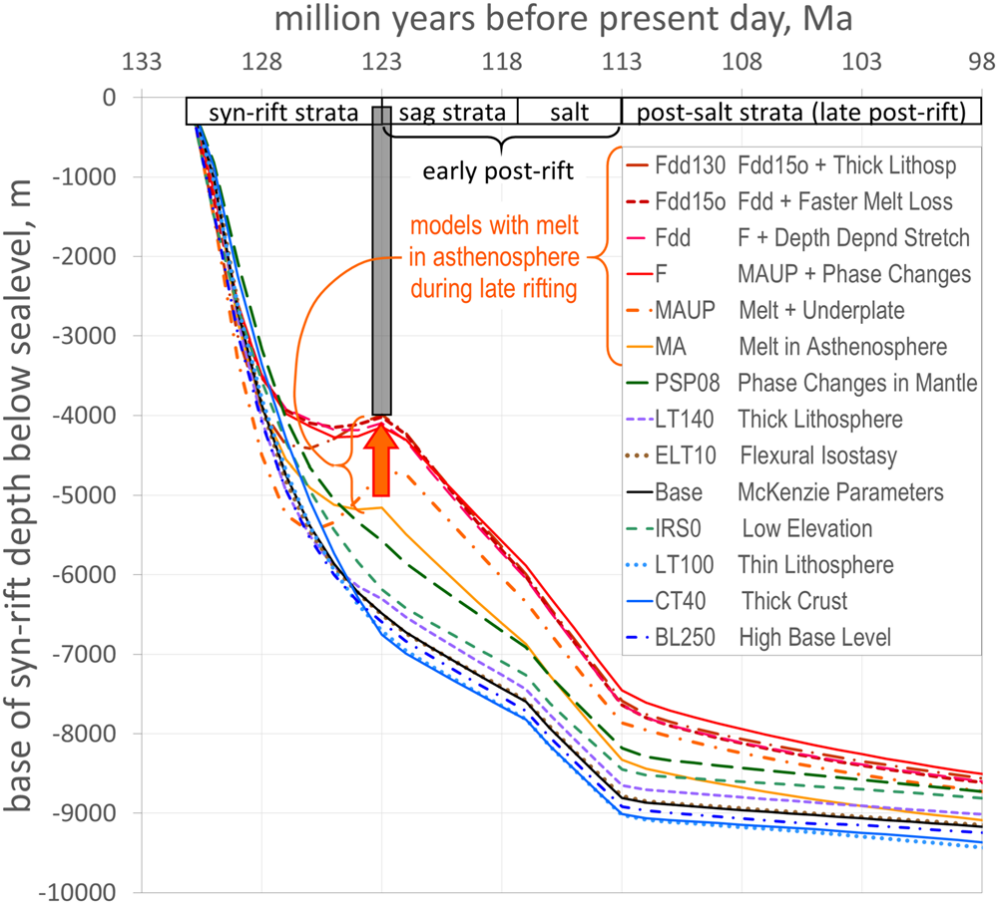
Graphs comparing subsidence histories of a 131 million year old surface (base of rift) for different models of the margin shown in Fig. 1 at a position 40 km inboard of breakup point. All models converge to a subsidence depth of 11,600 m at 0 Ma. The grey bar indicates the thickness of syn-rift sediment at the time of breakup. Any difference in depth between the grey bar and a specific subsidence line at 123 Ma represents the water depth (unfilled accommodation space) which, if significant, contradicts the palaeo-bathymetric evidence of contemporaneous erosion and subsequent shallow water sag and salt deposits. The red arrow indicates end-of-rift rebound in models with melt and underplates.

The results of the examples noted above are summarized in Table 1. Models illustrated in Figs 1a and 2 are those which best represent a single concept or those which in combination with others come close to matching the stratigraphic thicknesses in our original interpretation (Fig. S3). We start with models based on standard McKenzie-type assumptions (the base case) and then proceed to test different water levels, sediment fill and preservation scenarios, alternative crust and lithosphere thicknesses, changes in flexural isostasy, different elevations of the initial rift, as well as the effect of mineral phase transitions in the mantle, underplating and depth dependent stretching (where the amount of thinning in the crust and mantle lithosphere differs). Finally, we introduce melt in the asthenosphere during rifting (Model MA), combined with underplating and phase changes (Models MAUP and F), and test the effect of depth dependent stretching (Models Fdd and Fdd15o) and thicker lithosphere (Model Fdd130). Other models, including alternative ages and depths of horizons, different durations of rifting and mantle exhumation are included in Supplementary Information (Figs S1 and S2), largely aimed at showing sensitivities to different interpretations.

There are three main elements in judging whether the models provide valid explanations for the elevation-subsidence paradox at rifted margins: (i) similarity in thicknesses of sediment infill (stratigraphic fit); (ii) water depths corresponding to the actual depositional environments (palaeo-bathymetric match); (iii) a relatively flat sediment surface at breakup, appropriate for the development of shallow water sag strata and salt across the entire basin (topographic match). The second and third requirements are the most challenging^37^. Achieving shallow water depths during deposition of sag and salt is difficult, even allowing for uncertainties +/−250 m in global sea-level. More significantly, a flat sediment surface is not achieved with any of the models by themselves (Fig. 1a and Fig. S1 in Supplementary Information). The problem comes from the fact that the upper layers in both the sag and the salt extend monotonously over the entire basin^6,13,32–34,37,38^. The base case model summarizes the problem where, by the time breakup occurs at 123 Ma, the sediment surface of the thinned continental plate has sunk to around 3000 m below sea-level in the outboard region (Fig. 1a), with the amount of subsidence directly related to the degree the crust is stretched^1^. In reality, breakup is marked by subaerial erosion across most of the central South Atlantic region^4^ and the underlying syn-rift strata are continental and relatively thin whereas, for 10 million years (My) after breakup, carbonates and salt were deposited at very high rates^6,32^. On other rifted margins, where siliciclastic sediments or volcanics predominate, sedimentation fails to keep up with post-breakup subsidence and the deepening of the sea appears almost catastrophic^7^.

To address the main question – how to keep the sediment surface close to sea-level across the entire basin during rifting – it is necessary to generate a source of buoyancy proportional to the degree of stretching. Subsequently, the buoyancy needs to be lost over a short period of time following breakup. This is where melt can play a role, assuming the amount present is related to the height the asthenosphere wells up (Fig. 1c, Movie S2 in Supplementary Information). Most other possibilities we have tested fail to match stratigraphy, palaeo-bathymetry or both (Table 1). Dry mantle phase transitions have a positive effect^15,16^ but are only sufficient to counteract one third of the excess subsidence in the outboard region (Figs 1a and 2). However, when phase changes are combined with melt retention during rifting, the water depth at breakup is reduced to no more than a few hundred metres with a good fit to present day stratigraphy (final models pre-fixed F in Table 1 and Fig. 1). In view of uncertainties in sea-level, in the exact ages and thicknesses of strata and in other elements of interpretation, the models utilizing melt and phase changes together (e.g. Fdd, Fdd15o and Fdd130, Fig. 1a) are regarded as having successfully matched the subsidence history of a rifted passive margin (Fig. 2). In contrast, a McKenzie-type model (base case, Table 1) fails to explain thin syn-rift, elevated breakup and rapid post-rift sag.

The parameters used to achieve the match in models Fdd, Fdd15o and Fdd130 (Figs 1a and 2) are:

- Normal pre-rift thickness lithosphere of 120–130 km with 35 km crust and initial rift surface at 500 m above sea-level.
- The presence of melt in upwelling asthenosphere proportional to the height it rises above a depth of 90 km (when melting starts), reaching an average of 4–6% melt in a 40–55 km column for stretching factors of 3–5 (Fig. 1b) where the sag is thickest in the central part of the Kwanza Basin (Fig. 1c).
- Expulsion of melt exponentially after breakup so that ≤2% is left after 10 My in the central part.
- Sequential intrusion of sills of magma during rifting to create an underplate at Moho level, thickness proportional to the amount of melt produced in the asthenosphere (1–3 km thick in the central part, Fig. 1b).
- Phase transitions in the mantle as described for dry, fertile peridotite (R123) by ref.^16^ where, during late rifting, plagioclase- and spinel-bearing lherzolites predominate.
- Minor differential thinning between crust and mantle lithosphere.

Together, these produce c.2600–2800 m of uplift at the rifted edge of the continent relative to the base case at breakup, as well as the desired rapid post-rift sag. The mantle densities resulting from the melt models are similar to those from gravity measurements in the East Africa Rift System^18,22^, reaching a minimum of c.3100 kg.m^−3^ (Movie S3 in Supplementary Information). Larger amounts of melt, significantly different rates of melt loss, thicker intrusions and more severe phase transitions^15^ do not fit the stratigraphy and assumed bathymetry. Also, by themselves, no other effects are sufficient to stop the surface of the plate sinking to oceanic depths at the point of breakup (Fig. 1a).

It is worth noting that the thicknesses of underplates produced in models Fdd, Fdd15o and Fdd130 (Fig. 1b) are similar to those of high velocity, high density bodies seen at the base of many rifted basins^41^, including the South Atlantic^42,43^. They are generally reflective with a layered appearance, atypical of serpentinized mantle^44^ and are generally interpreted as underplates or layers of mafic and ultramafic intrusions although ref.^45^ leaves the question open. However, the model underplates thicken towards the basin centre or ocean (similar to modern rifts and some seismic reflection and gravity interpretations in Santos-Campos-Kwanza^6^) whereas interpretations of seismic refraction data in the South Atlantic often show them tapering out towards the ocean^45^. Nonetheless, once other effects such as phase changes are incorporated into the models, the difference in bathymetry with or without underplates is less than 150 m in terms of water depth at the end of rifting (Fig. S1 in Supplementary Information). In other words, the overall effect on subsidence of underplates is relatively small: we include them more to show that some melt makes its way out of the asthenosphere by breaking through the lid of mantle lithosphere in the form of intermittent magmatic intrusions.

One unexpected outcome of models incorporating melt is a rebound effect at the end of rifting (Fig. 2), where uplift from mantle buoyancy exceeds subsidence caused by crustal thinning. This may provide an explanation for the long-debated end-of-rift erosion surfaces or unconformities observed in most rift basins^3,4^.

## Discussion

There are many uncertainties in interpretations of rifted margins but there is general agreement that water depths and subsidence rates around the time of breakup do not fit with the standard stretching-cooling model. The models presented in Figs 1 and 2 and additional data in Supplementary Information show that melt-related density changes in the mantle provide an alternative way of creating elevation and subsidence anomalies, in addition to those caused by dynamic topography, phase changes and depth dependent stretching.

Nonetheless, many researchers have already addressed the rift-sag-salt thickness and water depth issues in the central South Atlantic region. One explanation is that the sediment surface was controlled by a local lake level which was drawn down by more than 1000 m below global sea-level when late rift and early post-rift strata (including salt) were deposited^32,37^. As well as there being no evidence for catastrophic flooding when global sea-level was re-established^6,34^, this model is difficult to reconcile with the most up-to-date plate reconstructions^46^ showing that an ocean around 200 km wide had been established by the time that salt was deposited in the late Aptian, 115 Ma (Movie S4 in Supplementary Information). These reconstructions are supported by palaeontological evidence for marine influence^33,35^. Nonetheless, we test the draw-down idea with two models UFB (underfilled basin) and BL-250 (base-level 250 m below global sea-level) and others in Supplementary Information but none of these come close to achieving a good stratigraphic match or the correct depositional water depth conditions (Table 1 and Fig. 1a). Therefore, as well as testing different tectonic and stratigraphic scenarios, the type of basin modeling reported here can be used to help verify or reject plate reconstructions.

Depth dependent stretching has also been proposed as a way of dealing with the apparent imbalance between syn-rift and sag thicknesses^10,40^, one idea being that rifting continued with stretching of the mantle lithosphere after movement on extensional faults in the crust had ceased, making the thick sag and salt intervals part of the syn-rift sequence^13,14,43^. We find that by itself, even a relatively large difference in the amount of thinning of the crust versus the mantle lithosphere does not have sufficient effect on subsidence (e.g. Model DD, Table 1) but when combined with melt and phase changes, moderate amounts of differential thinning help smooth the water depth and subsidence profiles (e.g. Models Fdd, Fdd15o, Fdd130).

Other researchers have suggested extreme degrees of depth dependent stretching occurred so that the crust was thinned to effectively zero causing the mantle to be exhumed and possibly serpentinized by hydration via crustal-scale faults^13,14,43^. Mantle exhumation is proven to occur only in regions of very slow spreading (<15 mm per year) such as Iberia-Newfoundland, where cooling inhibits melting, not in rifts with normal extension rates (>20 mm per year) as was the case in the central South Atlantic (Fig. 1a in ref.^6^). Although numerous wells have now penetrated the syn-rift section in the outermost part of the rifted margins of Brazil, Angola and Gabon, to the best of our knowledge none have yet found any evidence of exhumed serpentinized mantle and more recent geophysical interpretations are also tending to rule out its presence^37,44^. For completeness, we have explored scenarios of mantle exhumation and serpentinization (Models MntlEx and MntSerp, Table 1). Excess subsidence of syn-rift, sag and salt horizons occurs when the crust is thinned to effectively zero during mantle exhumation and there is a very poor match to stratigraphy and palaeo-bathymetry (Fig. S1 in Supplementary Information). To overcome the density of normal lithospheric mantle, extreme degrees of mantle serpentinization would be necessary, a process which can be simulated in TecMod. Serpentinization is the metamorphic reaction that turns a dry peridotite into a wet serpentinite by influx of seawater. The reaction is associated with a decrease in density and the release of latent heat. It therefore affects the structural and thermal solution. A prerequisite for serpentinization is that the entire crust becomes brittle so that deep faulting can take place, providing the pathways for seawater to reach and react with cold mantle rocks. In order to resolve mantle serpentinization processes it is therefore necessary to track the rheological evolution of the extending lithosphere. Our modeling shows that syn-rift and early post-rift sedimentation suppresses crustal-scale faulting and therefore serpentinization is severely limited with only a small effect on subsidence (Model MntSerp, Table 1 and Fig. S2 in Supplementary Information). Furthermore, even if significant amounts of serpentinite were to form, late syn-rift and early post-rift temperatures are too high for serpentinite to remain stable. Our models effectively rule out the possibility that mantle exhumation and serpentinization occurred in the central South Atlantic region. This result is likely to hold true for rifted margins with normal rates of stretching and sedimentation.

### Retention of melt beneath continental rifts?

Where continental rifting occurs, the lithosphere thins and the asthenosphere wells up. Due to pressure reduction, the asthenosphere intersects a line defining the onset of melting (the solidus) for peridotite^47^: initially the wet solidus, producing a very small amount of water-enriched melt (c.2%, ref.^12^), which dries the asthenosphere. Then at depths of around 90 km the asthenosphere reaches the dry solidus to produce significant amounts of melt^11,21^, preferentially from garnet/spinel and clinopyroxene^48^. In regions of normal mantle temperatures, ≥10% melt is produced for 1 GPa decompression of dry lherzolite^47^, equivalent to asthenosphere rising 30 km. Mafic melt is 15–20% less dense than parent peridotite at the same depth^28^ so that for every 1% of melt the density of the mantle decreases by 0.2% or 6 kg.m^−3^ which, with isostatic compensation, would account for 150 m of uplift if the melting zone is 70 km thick^20,49^. There is no doubt that significant amounts of melt are generated during rifting and not all of it reaches surface, even on volcanic margins^5^. There is however disagreement in how permeable the asthenosphere is to melt and therefore how much is retained in the lithosphere (discussed in Supplementary Information). Some of the uncertainty also touches on the debate concerning the importance of plumes and other mantle anomalies^18,20,21^.

A model of high melt retention in the asthenosphere is most likely not appropriate beneath oceanic spreading centres. It is generally accepted that the melt responsible for mid-ocean ridge basalts (MORB) has a relatively short residence time in the mantle^50^. Enrichment of MORB in short-lived uranium decay series isotopes such as ^230^Th and ^226^Ra suggests that melt beneath mid-ocean ridges becomes interconnected at low porosities (e.g. 0.1%) and is expelled upwards relatively quickly (e.g. at rates in the order of 10 m per year)^27^. Nonetheless, dynamic models of seafloor spreading show that it is difficult to maintain the required conditions of high permeability in the upper part of the asthenosphere without extracting more melt than the isotope data supports^51^. One explanation for this apparent contradiction is that the isotope data record an initial low viscosity, low density, volatile-rich melt generated at depth^48^. This early melt ascends to where the majority of melting and extraction occurs in the upper part of the asthenosphere^11^, thus contaminating the isotopic signature^51^. Porosities of 1-≥5% are expected in this upper zone based on theory and various geophysical measurements around mid-ocean ridges^27,52^. Higher amounts of melt have been interpreted in the asthenosphere beneath some continental rifts^30,49^ (Table 2), similar to our melt models prior to breakup (Fig. 1). The obvious difference to oceanic rifts is the presence of a relatively impermeable lid of continental mantle lithosphere which is known to trap melt at the top of the asthenosphere. Thus, uranium isotope and trace element data in continental rift basalts show evidence of slow upwelling of asthenosphere (e.g. 10–20 mm per year) and a lithosphere mantle signature suggesting melt is pooled at the base of the plate, with only periodic extraction of magma by intrusive or fracture-related processes^19^. The amount of melt generated during rifting can be calculated^26^, the question is rather how much is retained in the asthenosphere before the lithosphere lid breaks.

The number of parameters affecting melt connectivity, permeability and migration in the asthenosphere are large, including (i) whether there is an element of convective or active upwelling and, if so, its thermal and chemical effect, (ii) the shape, orientation, concentration and connectivity of melt-filled pores, (iii) the question of whether textural equilibrium is achieved between crystal grains, (iv) the importance of interfacial tension and other percolation thresholds in suppressing melt migration, (v) the viscosity of the melt and (vi) the permeability of the asthenosphere and the overlying mantle lithosphere^11,23,27,29,47–49,51,53,54^. There are currently too many uncertainties to model the various interactions and it is therefore difficult to estimate from theory the amount that is retained. Instead we turn to empirical evidence, specifically geophysical measurements indicating concentrations of melt present below present day rifts (Table 1).

Interconnected melt is proven to exist in the upper mantle in areas such as the East Africa Rift Valley and the Transantarctic Mountains based on anomalous electrical conductivities and seismic velocities^24,30,31,49,55,56^ but the differences in interpretations are significant, with amounts of melt ranging from c.0.5% to >15% (Table 2). For example, there is a five times difference between ref.^54^ and ref.^57^ in the percentage of melt estimated to cause a unit reduction in S wave seismic velocity, largely related to the assumed orientation and aspect ratio of melt inclusions^49^. Better constraints are now coming from electromagnetic measurements. A large increase in conductivity occurs when melt pores become connected, a situation which may require >10% melt in peridotite containing pyroxenes^58^. This amount of melt is an order of magnitude higher than typically used in numerical models^19^ but is similar to interpretations of magnetotelluric data from the upper mantle beneath some active rifts^30,49^. Although there is no consensus at present, we suggest that high melt retention is possible beneath continental rifts for two reasons: (i) where large amounts of melt are being generated within upwelling asthenosphere, porous flow may be inhibited because the mantle comprises multi-mineralic lherzolite which is not texturally equilibriated^54,58,59^; (ii) overlying continental mantle lithosphere forms an impermeable lid prior to breakup^20,23,25^. This can lead to the development of a zone of melt-impregnated mantle at the top of the asthenosphere which gains thickness as melt drains downwards by capillary action once the pores become connected^53^. Further details of the parameters affecting melt retention and migration are presented in Supplementary Information, although it should be reemphasized that the uncertainties in the physical and chemical state of the asthenosphere beneath continental rifts are large. Nonetheless, our conclusion is that it is possible that asthenosphere retains 5–16% melt during the latter stages of continental rifting prior to breakup^30,49^, significantly more than the <1–3% melt expected below mid-ocean ridges^11^ where there is no mantle lid. In our melt models, we actually use average amounts at the lower end of this range (≤8%, Fig. 1c, Movie S2 in Supplementary Information).

**Table 2:** Interpretations of the amount of melt present in the upper mantle below the East Africa Rift System based on various geophysical techniques.

Reference	Region	Rift setting	Main geophysical method used	Partial melt zone		Amount of melt calculated in upper mantle			
				Depth range, km	Width, km	Author range	Mini-mum	Maxi-mum	Implied melt thickness, km
Hammond & Kendall^49^	Dabbahu Rift, Afar	continent transition	P and S wave velocities and anisotropy	<26–90	c.150	6-≥15%	6%	15%	3.0–9.8
Desissa *et al*.^30^	Dabbahu Rift, Afar	continent transition	magnetotellurics	8–36	30	≥13%	6%	15%	≥3.6
Stork *et al*.^56^	Dabbahu Rift, Afar	continent transition	P wave velocities	>30-c.75	100–200	3%	2%	6%-11%	c.0.7-c.3.8
Gallacher *et al*.^55^	Afar Depression	continent transition	S (Rayleigh) wave imaging	c.20-c.120	c.100	0.3–0.5%	0.3%	6.4%	0.1–5.1
Hammond *et al*.^31^	Afar Depression	continent transition	P and S wave velocities	75–200	50–200	—	0.5%	1.5%	0.6–1.9
Rychert *et al*.^20^	Afar Depression	continent transition	S to P wave conversion imaging	0–75	50	c.1%			0.8
Gallacher *et al*.^55^	Ethiopian Rift-Afar	continental	S wave velocities	c.20-c.120	100–150	0.3–0.6%	0.3%	4.1%	0.2–5.1
Hammond & Kendall^49^	Main Ethiopian Rift	continental	P and S wave velocities and anisotropy	<35-c.85	c.100	2–7%			0.8–3.9

The minima and maxima are based on graphs and data in refs^49,54,57^ and other references in Supplementary Information.

Melt collecting at the top of the asthenosphere can develop into a magma reservoir for intrusions which periodically penetrate the continental mantle lithosphere. The magma is transported within discrete plutonic bodies, usually assisted by fractures and shears^23^ rather than by porous flow, and may ultimately form sills and underplates at the base of the crust^18,22,41^.

After breakup, the mantle down to c.90 km below the rifted margins (now inactive) is largely devoid of garnet-spinel-plagioclase and clinopyroxene, these minerals having already been lost to melt. The depleted asthenosphere consists predominantly of olivine-rich, texturally-equilibriated harzburgite-dunite which wettens easily so that the remaining melt is interconnected at low porosities (<1–3%) and migrates relatively quickly to the base of the lithosphere. Provided the melt does not solidify, it will then flow up-dip towards the edge of the continental plate to be incorporated in thick oceanic crust created at the new spreading axis. As the depleted asthenosphere cools, it is gradually accreted to the base of the continental plate to form new lithosphere together with melt which has solidified before reaching the oceanic domain.

One of the implications of our models is that melt can have residence times of more than 1 My in the asthenosphere where stretching factors are high (>3) provided the continental lithosphere remains intact (Movie S2 in Supplementary Information). We feel that this is an area that deserves further attention, particularly with reference to uranium series isotopes.

## Final Remarks and Conclusions

Thin syn-rift strata and subaerial to shallow water conditions at breakup followed by rapid post-rift sag may in part result from melt trapped in upwelling asthenosphere which is suddenly released when the continental mantle lithosphere breaks. In contrast, mantle exhumation or continued extension during sag deposition or desiccation of the basin either make the subsidence problem worse or the stratigraphy cannot be matched. We cannot rule out that dynamic uplift and collapse is important but, like other effects, more than 1000 m is hard to justify. Changes in the density of dry mantle due to phase transitions have the right sort of effect but cannot match the subsidence history without an additional source of buoyancy. In some regions, a reasonable case can be made for early syn-rift strata being removed before the rift subsides below sea-level, but the question then shifts to what causes rifts to be elevated in the first place. We acknowledge that the amounts of melt we test seem high (up to 8% at stretching factors of >7.5) when compared to many existing models which use <1–2% retention^12,19^. Models with low retention assume the asthenosphere is predominantly olivine and in textural equilibrium^54^ and that melt can migrate to shallow levels relatively unimpeded^26^. In the continental domain these conditions may not be met. Higher amounts of melt are supported by recent geophysical interpretations at present day rifts (Table 2) implying that the asthenosphere may not be in textural equilibrium^29,49^, probably contains a significant amount of pyroxene^58^ and may be affected by grain-grain interfacial tension^53^. The biggest factor, however, is the presence of a relatively impermeable lid of continental mantle lithosphere^20,23,25^ below which melt will tend to pool prior to breakup^47^. Another way of considering the argument is with the question where does all the melt go prior to breakup if it is not retained in the upwelling asthenosphere? Several kilometres of magma seem to be missing on non-volcanic margins^40^ assuming the melt is lost from the asthenosphere. In this respect, it is interesting to note that in models where melt is retained in the asthenosphere (Fig. 2), buoyancy exceeds subsidence at the time of breakup, providing a possible explanation for the commonly observed end-of-rift erosion surfaces.

We treat the retention of melt in our rift models in a simple way, with the amounts based on empirical observations. More sophisticated models are currently hard to constrain because of uncertainties in chemical replenishment, melt connectivity, interfacial forces, permeabilities and migration rates in the asthenosphere and can easily take focus away from the scale of the problem shown in Fig. 1a: without an additional source of buoyancy, continental rifts subside to oceanic depths prior to breakup. If the amount of melt we model in the asthenosphere (Fig. 1c) is regarded as too high, something else is needed to counteract the isostatic effect of thinning the crust (Fig. 1b). Our work may therefore encourage the search for more elaborate solutions – either a better melting routine or an alternative way of keeping the plate elevated.

As a final remark, perhaps a less controversial way of framing the hypothesis is recognizing that some excess melt is present in the asthenosphere during rifting which at least in part will have a positive effect on elevation and subsidence. After breakup, a proportion of the excess melt will be incorporated in thickened oceanic crust seen at the edges of many oceans. There are wide ranges in interpretations of the amount of melt held in the asthenosphere during rifting but we conclude that melt will have some effect on subsidence. Our experiments indicate that 2600 m of subsidence or more can be deferred from syn-rift to early post-rift when combined with the effects of phase changes and underplating. The idea can be tested with better models and measurements of melt beneath rifting continental lithosphere.

## Methods Summary

### Experimental data

We use thermo-tectonostratigraphic basin modeling software (“TecMod”) to simulate the tectonic, thermal and subsidence history of a non-volcanic rifted margin based on a cross-section through the sedimentary fill of the northern Kwanza Basin of Angola (Fig. S3 in Supplementary Information). The cross-section is an interpretation of an east-west 2D depth-migrated seismic line (GX 2400) owned by ION GeoVentures extending 310 km from the coast to oceanic crust at a water depth of 3500 m. The interpretation uses chronostratigraphic, seismostratigraphic, lithofacies and tectonic information reported in refs^6,7,42^ and in Supplementary Information for the Angolan and Brazilian margins of the central South Atlantic, with the ages and palaeo-water depths of the key horizons based on well-seismic data from the Campos, Santos and Kwanza basins. The base of rift formed subaerially at 131 Ma, overlain by syn-rift strata comprising continental siliciclastic sediments deposited above sea-level ending with breakup at 123 Ma. Rifting is followed by remarkably thick early post-rift strata with lacustrine mudstones and carbonates of the “sag” interval from 123–117 Ma and salt (predominantly halite) from 117–113 Ma. These were deposited in a narrow sea or lake with water depths remaining shallow by virtue of very high sedimentation rates (up to 1 mm per year): both carbonate production and evaporite deposition were able to keep up with rapid subsidence.

Taking into account variations in global sea-level and uncertainties in interpretation, a close palaeo-bathymetric match is assumed to have been achieved in models with <500 m difference between the modeled top of the sediment surface and sea-level when sag and salt are deposited. Although salt is an excellent water depth indicator, it does add structural complication. To simplify modeling, local variations in sediment thickness related to vertical salt movement have been ignored by continuing horizons through diapirs when the seismic section was digitized (see Fig. S3 in Supplementary Information) with slight adjustments to ensure the correct amount of salt is maintained. Lateral flow of salt occurred during and soon after deposition but this served to fill accommodation space in outboard areas by gravitational spreading^34^ so it can be treated as redistributed sediment similar to sandstone or shale, in line with the time resolution of the model. In addition, it is not certain that the salt was deposited at sea-level as it is possible that the level of the lake in which the salt was deposited was drawn down by evaporation^32,40^, an idea we test in models BL-250 and BL-1k (Table 1 and Figs S1 and S2 in Supplementary Information).

### Modeling method

We utilize a modified version of the commercial basin modeling software TecMod, which uses an iterative, inverse finite element algorithm to reconstruct the stretching, subsidence and thermal evolution of rift basins. It takes the observed stratigraphy as input and automatically computes the best-fitting structural and thermal solution using an iteratively-refined forward model locally based on pure shear kinematics and resolves flexural isostasy, sedimentary fill, compaction and heat transfer without boundary conditions in the lithosphere and asthenosphere. The technical details of TecMod are described in refs^9,39^ and in Supplementary Information. We have adapted the software to the special needs of this study by separating the properties of the mantle lithosphere and asthenosphere and introducing a new parameterized melting routine. The effect of the melting routine can be seen in Fig. 1c. The models have been tuned by calibrating to specific observations in present day rifts and rifted margins including mantle conditions^12,17,25^, surface elevations^18^, interpreted melt percentages (including high amounts^30,49^) and subsidence histories^6^. We have also included the option of introducing underplates in the form of mafic sills at the base of the crust, proportional to the amount of melt retained in the asthenosphere (≤25%) during every time step. For the Kwanza model reported here, this results in an underplate 5 km thick at the point of breakup tapering to 0 km towards the inboard edge of the rifted margin (Fig. 1b). We assume average global sea-levels throughout except for models where the basin is desiccated during rifting (e.g. BL-250 in Table 1).

The base model assumes that the crust is initially 35 km thick, comprising 80% granitic upper crust and 20% gabbroic lower crust, and the lithosphere is 120 km thick, has zero elastic thickness and is subjected to depth uniform extension in the form of pure shear. The land surface lies at 500 m above sea-level at the start of rifting. The asthenosphere has a reference density of 3300 kg.m^−3^, the same as lithosphere, and the mantle contains neither melt, nor intrusions. Subsequent models were tested with different crustal thicknesses, lithosphere thicknesses, asthenosphere densities, surface elevations, sedimentation rates and base levels, as well as with and without flexural isostasy, mineral phase changes in the mantle, various degrees of melt in the asthenosphere, underplating at the base of the crust (intrusion of sills) and depth dependent stretching (Table 1). Those which have significant effect on reducing subsidence during rifting are melt in the asthenosphere and phase changes which together are sufficient to keep the surface of the plate close to sea-level until breakup whilst matching stratigraphic thicknesses (models prefixed F, Table 1). Under these conditions, an acceptable model is achieved with uniform lithospheric extension (e.g. Model F, Table 1 and Fig. 1) but minor depth dependent stretching allows for a better stratigraphic match (e.g. Model Fdd), also smoothing out the effects of syn-rift faults. Extreme depth dependent stretching, where the lithospheric mantle was removed (Type II rifted margin, ref.^10^), did not reach a solution as lithosphere composed only of crust proved too light for the deposition of late syn-rift and early post-rift sediment.

We do not account for subsidence and uplift related to volume changes in the mantle, these tending to exaggerate the isostatic effects of melt and phase changes. Also, other minor factors have been ignored, including:

- Changes in the fertility of peridotite and therefore its density as melt is extracted^17,19^.
- Minor differences in density due to the presence or absence of water and other volatiles in peridotite^48^.
- Compaction and subsidence resulting from changes in volume associated with loss of melt and crystallization of melt.
- Other forms of metamorphism.
- Heterogeneities in sediment properties.

In addition, the dynamic effects of active mantle plumes are not included in our models. Dynamic uplift and subsidence can be on the scale of 200-≥1000 m^3,6^ but they are likely to be basin specific rather than a generic effect associated with all breakup events. Possible enhanced convection caused by phase changes and melt^55^ is also ignored.

For every 1 million year step, each model records the depth of strata relative to global sea-level which, for simplicity, we assume is fixed. In addition to assessing the stratigraphic match of the model to the original horizons at present day (Fig. 1b), the most informative results are profiles of the depth of the top of the sediment surface at any specific point in time (e.g. Fig. 1a), the temperature, density and percentage of melt in the lithosphere and asthenosphere (e.g. Fig. 1c and Fig. S4b in Supplementary Information) and separate stretching factors for crust and lithospheric mantle (Fig. S4c,d in Supplementary Information). In addition, the subsidence history of any surface can be viewed (e.g. Fig. 2), as well as colour-coded cross-sections of, for example, palaeo-water depths of all strata deposited in the model (Fig. S4a).

### Models with melt

The height that the asthenosphere rises during rifting is used to determine how much melt is generated and retained. In most of these models, we assume melting starts in the asthenosphere at a depth of 90 km with the amount increasing linearly to an assumed maximum of 16% should it reach 20 km (stretching factors >7.5), giving a maximum average melt of 8% through an asthenosphere column of 70 km (depth of 90–20 km). This is equivalent to a maximum total melt column of 5.6 km, feasible based on selected interpretations of electrical conductivity, seismic velocities and gravity which show up to 12 km in the upper mantle below continental rifts (Table 2). It should be noted that our models only show such high degrees of lithospheric stretching and melt close to the point of breakup (<35 km) and then only during the last 1 My of rifting. In the main part of the Kwanza Basin, stretching factors of 3–5 have developed by the end of rifting with an average 4–6% melt in a column of asthenosphere 40–55 km high, representing 1.6–3.3 km of magma, in line with many present day rifts (Table 2).

In theory it is possible to calculate the amount of melt produced in upwelling asthenosphere and this theory has been effectively applied in the relatively simple situation of mid-ocean ridges^11^. It is more complicated to do this in a continental rift setting^12,19^ and it is particularly problematic to calculate how much melt is retained in the asthenosphere because of uncertainties in several of the important parameters. We have tested melt functions using different published solidi^11,19,25,48,58^ but the models are strongly influenced by the evolving thickness and thermal structure of the lithosphere. Furthermore, we cannot account for the effect that flow patterns have on the chemical composition of the asthenosphere. We instead revert to empirical data on the amount of melt interpreted beneath some modern rifts (3–16% melt over several tens of kilometres, Table 2), the thickness of intrusions prior to breakup (underplates 3–6 km thick), the thickness of extrusive rocks associated with breakup on volcanic rifted margins (≥6 km) and the thickness of oceanic crust (≥6 km). In addition to melt retention in the asthenosphere, we assume in most of our melt models that some magma is also intruded in the lithosphere as sills or underplating at the base of the crust (Fig. 1b). Again, based on empirical observations^6,42,43^, we choose a gabbroic sill to be intruded every 1 million year of rifting with a thickness equivalent to 25% of the amount of melt retained directly below in the asthenosphere. In the Kwanza Basin example modeled here, this results in a maximum thickness of underplate of 5 km at the point of breakup after 8 My of rifting. This tapers to 0 km towards the inboard edge of the rifted margin (Fig. 1b). The difference in water depth caused by these intrusions is not huge (e.g. <400 m between Model M and Model Mup, Fig. 1a and Table 1) but we feel that it is important to include the effect because underplates and sills are commonly observed beneath rifted margins^5,41–43^.

We assume that lithospheric extension stops immediately after breakup, associated with the formation of a mid-ocean ridge. The time over which most of the melt is lost from the asthenosphere (c.10 My) is affected by when the asthenosphere stops flowing, the time for textural equilibrium to become established and for melt to become fully interconnected, by the pressure gradient driving melt migration, the slope at the base of the lithosphere and the distance to the nascent spreading centre, as well as the delaying effect on cooling caused by latent heat of crystallization and by the increasingly sluggish rate of migration of melt as porosity decreases. Again, these parameters are difficult to constrain but from observations of the thicknesses of early post-rift strata^6,13,38^, interpreted changes in water depth^6,7^ and the temporal nature of massive post-breakup volcanism and thickened early oceanic crust^5,47,60^ we suggest that the amount of melt probably decreases exponentially over time with no more than a few percent left in the asthenosphere by 10 My after breakup. Thus, we choose a rate of either 10% melt loss every 1 My (i.e. only half is left after 7 My and <3% melt remains after 10 million years, e.g. Model MA) or 15% melt loss every 1 My (i.e. less than half is left after 5 My and <2% melt remains after 10 My, e.g Model Fdd15o).

### Effect of phase transitions

The marked reduction in pressure versus temperature as the lithosphere thins and asthenosphere rises causes phase transitions in the mantle^15,16^, initially garnet (+olivine) changing to spinel (+orthopyroxene) at depths of around 51 km and then spinel (+pyroxenes) to plagioclase (+olivine) at around 26 km, leading to a decrease in density of c.30 kg.m^−3^ and c.55 kg.m^−3^, respectively. These effects become important at stretching factors of ≥2.5, accounting for a decrease in average density of mantle lithosphere in the order of 10–20 kg.m^−3 ^^16^. After rifting, there is a reversal in phase transitions due to cooling leading to an equivalent increase in density and enhanced subsidence. The calculations of refs^15,16^ can be utilized in TecMod: models using the latter work best (e.g. Model PSP08, Table 1 and Fig. 2) as we were unable to match the stratigraphy using the former.

## Electronic supplementary material

Supplementary text and figures S1-S4

Supplementary Movie S1

Supplementary Movie S2

Supplementary Movie S3

Supplementary Movie S4, plate reconstruction
