# Supplementary material for: Melt-induced buoyancy may explain the elevated rift-rapid sag paradox during breakup of continental plates
Source: Sci Rep. 2018 Jul 3;8:9985. doi: 10.1038/s41598-018-27981-2 (PMC6030112; doi:10.1038/s41598-018-27981-2)
Supplement: Supplementary file 1 — Supplementary text and figures S1-S4 [file 41598_2018_27981_MOESM1_ESM.pdf]

Supplementary Information for

Melt-induced buoyancy may explain the elevated rift-rapid sag paradox during breakup of continental plates

David G. Quirk[1] & Lars H. Rüpke[2]

[1]Manx Geological Survey/University of Manchester, Gammel Mønt 31, 1117 Copenhagen K, Denmark.
[2]GEOMAR Helmholtz Centre for Ocean Research Kiel, Wischhofstraße 1-3, 24148 Kiel, Germany.

Correspondence and requests for materials should be addressed to:
D.G.Q. (email: dgquirk@outlook.com) or L.H.R. (lruepke@geomar.de).

Timing of breakup relative to the sag and salt intervals in central South Atlantic

Disparities in the rates and timing of subsidence at rifted margins from those predicted by McKenzie's rift-cooling model[1] are expressed as thinner than expected syn-rift thicknesses, greater than expected early post-rift thicknesses and subaerial-shallow water conditions at breakup rather than deep water[2-26]. These issues are well known in the central South Atlantic region where, for example, the early post-rift sag and salt section is >4 km thick in northern Kwanza and composed of shallow water-emergent carbonates and evaporites. This has led some researchers to argue that the sag and salt intervals (Aptian, 123-113 Ma) are actually late syn-rift[3,15,27]. However, neither water depths nor basin geometry really support the idea (e.g. Model tR113, Figs.S1 and S2). In particular, the absence of extensional growth faults in the sag[2-5,10,15,27] (Fig. S3) and the geometric requirement of large trans-continental shears in plate reconstructions to accommodate late breakup[24,25,28], leads us to strongly favour breakup at c.123 Ma (pre-sag). This interpretation is supported by reported ages of syn-rift, sag and mid-ocean ridge-type basalts in wells drilled at or close to the point of breakup in deep water Angola by Statoil, ConocoPhillips and Total in 2014-2015 (e.g. Dilolo-1, Jacare-1, Kamoxi-1, Umbundu-1) and by more recent plate reconstructions[5,29,30] (Movie S4).

Information on the ages of rifting

The best estimate of the onset of rifting is 131 Ma based on the oldest syn-rift sediments which are Hauterivian in age[16]. The precursor Paraná volcanics are precisely constrained by $^{40}Ar/^{39}Ar$ dating to 135 Ma +/- 1 My[31] and uniform layering within them shows that they were erupted before extension began. Information on the timing of the end of rifting (123 Ma) is reported in the main article and in Refs.[2,5,8,16,18,30].

Seismic interpretation

The nature of the seismic units in the central South Atlantic region has previously been described by Ref.[5]. The syn-rift interval is characterized by tilted and fanning reflection geometries. Strong parallel reflections at the base of the syn-rift interval correlate with the top of basalt (the major Paraná eruptive event, the precursor to rifting in the central South Atlantic region[8,31]). The top of the syn-rift interval is marked by an angular unconformity onto which flat-lying sag strata progressively overstep (Fig. S3). The top of the sag is a flat horizon above which the salt has a clear but chaotic seismic expression resulting from salt tectonic movement. The top of the salt is marked by a strong seismic reflection and is overlain by Albian carbonates characterized by growth, fault and fold geometries. These structures were formed during gravity spreading as the salt tilted towards the ocean as a result of enhanced subsidence in that direction[18].

Are anomalous subsidence patterns during breakup related to mantle plumes?

The aim of our work is to find a generic solution for the common problem of elevated breakup and subsequent collapse at rifted margins[6,11]. We acknowledge that the thermal and dynamic effect of a mantle plume can have a semi-regional effect[32-34]. However, we feel that this is not a universal explanation for the subsidence issue associated with McKenzie's model[1] during the breakup of continental plates. Nonetheless, one of us (D.G.Q., Ref.[5]) has previously promoted this idea for a region of the central South Atlantic lying south of the current study area (Fig.S3), where syn-rift strata are particularly thin. It is therefore feasible that anomalous uplift caused by mantle plumes may enhance buoyancy caused by melt and phase changes in the mantle but probably not along every rift margin.

Information on melt produced during rifting

In the accompanying article, we explore the idea that melt is retained in the asthenosphere during rifting and lost rapidly after breakup. That melt exists in the mantle beneath modern continental and oceanic rifts is recognized from an increase in electrical conductivity, a reduction in seismic velocities and a decrease in gravity[35-60]. The geophysical evidence for melt is also supported by theoretical models, experiments and geochemical data[61-73]. Unfortunately, the amount of melt is difficult to constrain because many properties of the asthenosphere such as interconnectivity of melt-filled pores and permeability are poorly constrained with large uncertainties in physical characteristics, including the shape and alignment of melt inclusions[40,43,74-77] and whether the peridotite is in textural equilibrium[78-80].

As geophysical data and geodynamic models grow, two different hypotheses have emerged on the amount of melt present beneath modern rifts, with continental and oceanic rifts often treated similarly.

1) A common view is that dihedral angles of melt formed in olivine-rich peridotite are significantly less than 60° [79,81] and the peridotite is texturally equilibriated[78] meaning that melt flows through the matrix at very low porosities. Consequently, the mantle will retain less than c.2% melt[35,62,70,83-86], migrating out of the source region faster than it is generated[67,87]. This view is supported by various geophysical interpretations of <1%-3% melt in regions of upwelling asthenosphere[43], more commonly in oceanic domains [48,50,54-56,59,88].

2) Alternative experimental work and theoretical models indicate that lherzolite can actually retain 5%-c.20% melt[79,80,89,90], consistent with other geophysical interpretations of c.5-16% melt in zones 30-100 km thick and 30->150 km wide in the upper mantle[44,46,47,91], including continental regions[52]. For example, Ref.[92], using magnetotellurics, and Ref.[77], using P-S wave velocities and anisotropy, have independently produced similar estimates of 6-15% melt over an interval 30-60 km thick in the upper mantle beneath the Dabbahu rift segment in Afar, Ethiopia, equivalent to a melt column of 3-10 km. Assuming these interpretations are valid, there are five potential reasons for high melt retention.
   a) Where peridotite is neither mono-mineralic nor equi-granular, melt does not become interconnected until porosities of somewhere between 5-20%[79,80,90].
   b) Textural equilibrium is difficult to establish in flowing mantle[77].
   c) Interfacial tension presents a significant barrier to compaction- and buoyancy-driven flow at porosities of less than c.20%, even where grain boundaries are wet[89,93].
   d) Viscous resistance to flow means migration can be slower than generation[86,94].
   e) Melt flowing between crystals or carried upwards in circulating asthenosphere is trapped by a viscous lid of mantle lithosphere which represents a permeability barrier[39,56,88,95,96], ultimately resulting in crystal fractionation[96-98].

One of the key points is that melting experiments on real peridotite show there is a drastic reduction in the interconnectivity of pores when minerals other than olivine are present[80]. As much as 21-29% basaltic melt can be retained in lherzolite containing less than c.80% olivine[90] up to the point of disaggregation[89]. A typical fertile lherzolite comprises 60% olivine, 20% orthopyroxene, 10% clinopyroxene and 10% garnet or spinel or plagioclase[99], so theoretically all the aluminium-rich phases, all the clinopyroxene and some orthopyroxene have to melt before the pores become fully connected. Thus, it may be that melt is retained in asthenosphere composed of heterogeneous lherzolite but will tend to flow out when it is mostly olivine, provided interfacial tension is overcome[89]. However, in the continental domain a more significant permeability barrier exists at the top of the asthenosphere in the form of the continental plate[95,96,98]. Magma can only ascend through the overlying lithospheric mantle in discrete, fracture-assisted intrusions rather than by porous flow[9,43,74,84]. These considerations offer a way of aligning models of low versus high melt retention beneath rifted margins.

– Prior to breakup, when rifting reaches a peak, large amounts of melt have been generated and a significant proportion is temporarily retained in the upwelling asthenosphere, initially because porous flow is inhibited in multi-mineralic, non-equilibriated lherzolite and thereafter because the overlying lithosphere forms a closed lid. The zone of melt-impregnated asthenosphere will tend to drain downwards by capillary action, a wetting profile driven by grain–grain tension.

– After breakup, the mantle down to c.90 km below the inactive rifted margins is largely devoid of garnet-spinel-plagioclase and clinopyroxene, these minerals having already been lost to melt. The depleted asthenosphere consists predominantly of olivine-rich, texturally-equilibriated harzburgite-dunite with a dihedral angle significantly less than 60° meaning most of the remaining melt is interconnected at low porosities (<1-3%) and migrates relatively quickly to the base of the lithosphere. Provided the melt does not solidify, it will then flow up-dip towards the edge of the continental plate to be incorporated in thick oceanic crust created at the new spreading axis. As the depleted asthenosphere cools, it is gradually accreted to the base of the continental plate to form new lithosphere together with melt which has solidified before reaching the oceanic domain.

With these processes in mind, we suggest it is possible that asthenosphere retains 5-16% melt during the latter stages of continental rifting prior to breakup[52,77,92], more than below mid-ocean ridges where there is no mantle lid. In our melt models, we actually use average amounts at the lower end of this range (those prefixed M and F in Figs. 1 and 2 in the accompanying article, Figs.S1 and S2 and Movies S1-S3).

It is also worth noting that the presence of melt beneath an extending plate is likely to cause periodic intrusions of sills or underplates into the lithosphere[97,], an effect which may partly compensate for thinning of the plate, allowing certain rifted margins to become surprisingly wide (e.g. Fig.8 in ref.[5]). Underplates are quite well documented beneath central South Atlantic rifted margins with interpreted thicknesses up to 6 km[14,25].

Based on the arguments above, we test the effect of relatively high melt retention in the asthenosphere. The idea is that beneath continental rifts up to breakup i) significant porosity is required for melt to become interconnected in the asthenosphere[76,79,80,89,90] and ii) the base of the continental lithosphere acts as a lid[39,57,88,95,96,98].

Once it becomes interconnected, melt migrates upwards through the asthenosphere by porous flow, controlled by compaction and buoyancy, viscosity, permeability and surface tensions[62,67,89,93,94,99]. The upward migrating melt will be impeded by continental mantle lithosphere and the zone of melt-impregnated asthenosphere will then increase in thickness downwards by capillary action, with wetting driven by grain–grain tension to give a smoothly decreasing melt profile[89,93], as used in our models (Fig. 1c in main article).

We choose to limit the average amount of melt in the asthenosphere to 8%, starting with 0% at 90 km depth up to a maximum of 16% at 20 km close to the point of breakup where the lithosphere is highly attenuated (stretching factor of >7.5, Figs. S4c and S4d). This is equivalent to a maximum column of 5.6 km melt. However, over most of the Kwanza Basin, where stretching factors are <5 (Fig. S4c), a column less than half this amount has developed in our models by the end of rifting. For comparison, more melt is involved in the construction of oceanic crust but here it ascends relatively quickly to a mid-ocean ridge, unimpeded by lithosphere. A total column of 7.5 km melt is needed for average oceanic crust[73,82,97] but as much as 12-24 km of melt may be involved during the formation of volcanic rifted margins[9,87,96].

We do not argue for unusual mantle conditions of temperature, only that a significant proportion of melt cannot migrate out of the asthenosphere until after breakup. In our models, the asthenosphere wells up passively while the lithosphere extends, a general assumption in most models of continental rifts[13,83]. In reality, the presence of melt in the asthenosphere may enhance convection due to buoyancy and a possible reduction in viscosity[60,61,83,99,100] although Ref.[101] suggests that viscosity may initially increase as the asthenosphere becomes drier with melting. If active upwelling or a mantle plume are associated with rifting, then higher degrees of melt are expected due to replenishment of fertile peridotite[96] or higher temperatures[64,82] which will lead to more volcanism at breakup[87].

Information on loss of melt after breakup

During rifting, significant columns of melt may be trapped beneath continental lithosphere until it breaks, after which it is likely to drain rapidly to the new volcanic ridge[87,94,102]. This may explain the release of huge volumes of magma over short periods of time forming thick crust on volcanic margins[82,96,97,102] as well as outer volcanic highs on non-volcanic margins[5,23,103]. In the North Atlantic Igneous Province, Ref.[104] suggests that a c.7 km column of melt per unit area was released to the crust in the 5 My up to breakup followed by 6-17 km soon after breakup, which we suggest was partly due to lateral migration of melt stored in the asthenosphere (cf. Ref.[105]).

After breakup, the asthenosphere beneath the rifted margins ceases to flow and textural equilibrium is re-established. Melt production also stops and porosity decreases causing the rate of melt migration to decline. The density of the mantle will increase as melt is lost, causing the rifted margins to subside. Based on empirical observations of post-breakup volcanism and thicknesses of early post-rift sag[5], we expect melt to be lost at an exponential rate with only small percentages left after 10 My, although the lithosphere continues to thicken and subside by cooling.

Basin reconstruction approach

TecMod is modeling software for lithosphere-scale basin tectonic, isostatic, stratigraphic, thermal modeling which accounts for the feedbacks between sedimentary, crustal, and mantle processes[106]. It is designed to produce automated 2D basin models using present day stratigraphy as input, from which the structural and thermal evolution of a rifted basin is automatically reconstructed. This is achieved through the coupling of a lithosphere scale forward model with an inverse algorithm for automated parameter optimization. This kind of algorithm allows the feedbacks and interactions between shallow sedimentary and deep lithosphere/asthenosphere processes to be investigated, which are difficult to predict without iterative modeling.

Given the number of physical processes involved and the range of parameters, the evolution of sedimentary basins cannot be uniquely reconstructed. The strategy employed in TecMod is scenario analysis performed on the (user-specified) geologically most likely end member cases, which yields a possible range in target parameters (mainly temperature, heatflow and related aspects though time and space). As demonstrated by Ref.[106], the actual fitting process of crustal and mantle stretching factors, water depth, sediment fill, thermal conditioning and isostatic adjustment can be fully automated by casting the problem in terms of a constrained optimization problem[107,108]. The goal function to be minimized is the misfit between observed and modeled stratigraphic data where additional constraints can be imposed (e.g. finite rifting durations, maximum stretching factors, weighting between different events). The inversion consists of an iterative search for the optimal set of crustal and mantle stretching factors and palaeo-water depth values, which minimize the chosen goal function[109,110]. The forward run requires an initial guess for stretching factors and water depth which are successively improved by iteration with the inverse algorithm according to the scheme described by Ref.[106].

The 2D model is locally based on pure shear kinematics and resolves simultaneously differential thinning, flexural isostasy, heat transfer, sedimentation, erosion and compaction on a Lagrangian finite-element mesh. The general modeling strategy is that the structural and

thermal solutions are in equilibrium after every simulation time step. This is achieved by resolving simultaneously for deep lithosphere and shallow sedimentary basin processes. During every time step, the entire computational mesh is deformed with the mantle and crustal stretching factors, new sediment packages are deposited and become part of the computational domain. Flexural isostasy is ensured by further movement of the mesh in the vertical direction.

Heat transfer occurs by advection and diffusion:

$$\rho_i c_i \left(\frac{\partial T}{\partial t} + v_x \frac{\partial T}{\partial x} + v_z \frac{\partial T}{\partial z} \right) = \frac{\partial}{\partial x}\left(k_i \frac{\partial T}{\partial x} \right) + \frac{\partial}{\partial z}\left(k_i \frac{\partial T}{\partial z} \right) + A_i \qquad (1)$$

where t is time and v_x and v_z are the velocities in the horizontal x- and vertical z-direction, respectively. In the employed Lagrangian reference frame, the numerical grid is deformed with the pure shear velocities so that the advection terms in equation (1) are automatically accounted for. In the case of differential thinning, re-meshing of the mantle ensures geometric consistency between the crust/sediments and the mantle portions of the modeling domain. ρ, c_i, T, k_i and A_i are density, specific heat, temperature, thermal conductivity and radiogenic heat production rate, respectively. The index i illustrates that the property is dependent on the local rock type. The thermal conductivity of the sediments is an effective material property that results from geometric averaging of the local matrix conductivity (km) and pore-fluid conductivity (k_w)[111,112]:

$$k = k_m^{(1-\phi)} k_w^{(\phi)} \qquad (2)$$

where ϕ is porosity. The temperature dependence of thermal conductivity is accounted for by assuming the following relations[113,114]:

$$\begin{aligned} k_w &= c_2 T^2 + c_1 T + c_0 \\ k_m &= k_m^0 e\,\mathrm{xp}\left(\frac{c_f}{T} - \frac{c_f}{T_0}\right) \end{aligned} \qquad (3)$$

with c parameters defined in Table S1. Crustal and mantle densities evolve during basin formation as functions of thinning, melt production, magmatic intrusion, heat transfer and sediment and water loading as well as phase transitions[115,116]. The resulting stresses on the lithosphere are projected onto a plate with an assumed elastic thickness and are compensated by isostasy [117-119]. The equation describing the vertical deflection of the top of the crust, w, due to flexural isostasy is:

$$\frac{\partial^2}{\partial x^2}\left(D \frac{\partial^2 w}{\partial x^2} \right) + \left(\rho_m - \rho_{in} \right) g \left(w + S \right) = q \qquad (4)$$

where x, D, ρ_m, ρ_{in}, g, and S are the horizontal coordinate, the flexural rigidity, the average density of the lithospheric mantle, the average density of the basin infill (compacted sediments), the acceleration due to gravity and the kinematic deflection due to necking. The differential load, q, represents differences in load relative to the load in the equilibrium state. The flexural

rigidity, D, is a cubic function of the effective elastic thickness (Te), which can be kept either constant or determined by the depth of a specified isotherm[120] or the thickness of the crust. The kinematic deflection S, i.e. the surface depression that results from stretching and not from isostatic compensation, is calculated using the crustal thinning factors and the depth of necking[118]. Both ends of the elastic beam are either matched to an analytical solution of a beam clamped at infinity or the modeling domain is extended laterally to minimize boundary effects. Although elastic thickness is believed to be close to zero during late rifting as the plate is thin and hot[12], flexural isostasy was nonetheless tested (e.g. Model ELT10, Table 1 in main article). The results were similar to those assuming elastic thickness is 0 km and the effect of flexure is therefore regarded as insignificant during breakup.

Sediments are incorporated in the thermal solver to resolve blanketing effects [121-124]. Sediment deposition is controlled by the time dependent water depth which comes out of the inversion (see below). For each time step, a local sedimentation rate is iteratively computed so that at the end of a time step, i.e. after sedimentation, compaction, isostatic compensation and flexure, the basin is filled to the pre-computed water depth. The deposited sediments are compacted using empirical compaction laws, specifically those published by Refs.[125,126] that have the form:

$$\phi_i = \phi_i^0 \exp(-c_i z) \qquad (5)$$

where ϕ is porosity, c is a compaction length scale and z the burial depth.

The boundary conditions for the thermal solver are user-specified fixed temperatures at the base and top of the numerical domain and zero horizontal heat flow at the sides utilizing a finite element method-based code. This allows the use of a body-fitting Lagrangian mesh strategy and ensures that all material contrasts are accurately resolved. The model and its individual components have been tested versus a number of analytical solutions including tectonic subsidence, thermal subsidence and steady state heat flow, with radioactive heat production rates decaying exponentially with depth.

Modifications and parameterization of melting and melt migration. We have introduced new features to TecMod in order to test our main hypothesis that melt retention is an important source of buoyancy during and shortly after breakup of continental plates. This has required i) tracking of the lithosphere- asthenosphere boundary (LAB) and accounting for possible compositional changes across it, ii) resolving melt generation, iii) melt migration, and iv) melt emplacement.

Tracking of the LAB has been implemented by assigning distinct rock properties to the oceanic and continental lithospheres and asthenospheres (Table S1). The employed Lagrangian mesh allows accurate tracking of the LAB during active syn-rift and passive post-rift deformation.

Simulations of decompression melting and melt migration continue to be numerically challenging. Models explicitly designed to resolve these processes typically use published solidus functions to solve for melt generation and the equations of compaction driven fluid flow to track the produced melts during their ascent towards the surface. We have deliberately not taken this approach. Models and observations on melt generation, retention and migration are still difficult to reconcile with geophysically inferred melt fractions in various rift settings as they are often

significantly higher than predicted by models. This and the considerable technical challenges associated with implementing melt migration into basin modeling software like TecMod has led us to derive a parameterization of melting and melt migration which allows us to investigate different scenarios that are consistent with geophysically inferred melt fractions.

We have parameterized decompression mantle melting by assuming that the degree of melting scales with the heights to which the LAB rises. The onset of melting is assumed to occur at 90 km depth, consistent with published solidus functions[65,69,94,98]. The concentration of melt is then assumed to increase linearly up to a specified depth of 20 km, where a maximum degree of melting of 16% is reached (average melt content of 8 %), which is consistent with observations at active rift zones and melt fractions calculated for adiabatic mantle upwelling[72]. Melt extraction is parameterized by assuming that the continental lithosphere is a permeability barrier for rising melts. As long as it is intact, melts are retained within the underlying asthenosphere. After breakup, loss of melt occurs exponentially at a prescribed rate of, for example, 15% every 1 My, meaning that more than half has been lost after 5 My and less than 2% melt remains in the asthenosphere after 10 My.

In addition, we have implemented the option to intrude igneous underplates. This allows us to explore scenarios in which a gabbroic sill of thickness equivalent to a specified fraction of the melt retained in the asthenosphere (e.g. 25%) is emplaced below the crust during every time step. For the Kwanza model reported in the accompanying article, this results in an underplate 5 km thick at the point of breakup tapering to 0 km towards the inboard edge of the rifted margin.

Finally, we use the models of Ref.[116] (specifically R123 peridotite) to test the effect that mineral phase changes in the mantle (both lithosphere and asthenosphere) have on the isostatic equilibrium of the plate.

Plate reconstructions

Recent plate reconstructions for the South Atlantic (Refs.[29,30] and Movie S4) use tectono-stratigraphic ages that fit with the comprehensive well and seismic data acquired by the oil industry in Brazil and Angola[5,8,16]. The first oceanic crust in the central South Atlantic area is interpreted to have formed around 123 Ma (early Aptian), correlating with the breakup unconformity at the base of the pre-salt sag strata and corresponding to the age of the outer volcanic high which marks the inner edge of oceanic crust in Angola[5]. The sag strata (123-117 Ma) and the overlying salt (117-113 Ma) were deposited inboard and thinly on top of the high, which is interpreted to represent the embryonic ridge[18]. The favoured plate reconstructions show a relatively simple breakup (e.g. Movie S4). These reconstructions are significantly different to the models of Refs.[24,28] where large amounts of trans-continental shearing are required in order to fit plates which remain attached for another 10 My, until the end of salt deposition. This shear movement is speculative, we believe resulting from an erroneous breakup age.

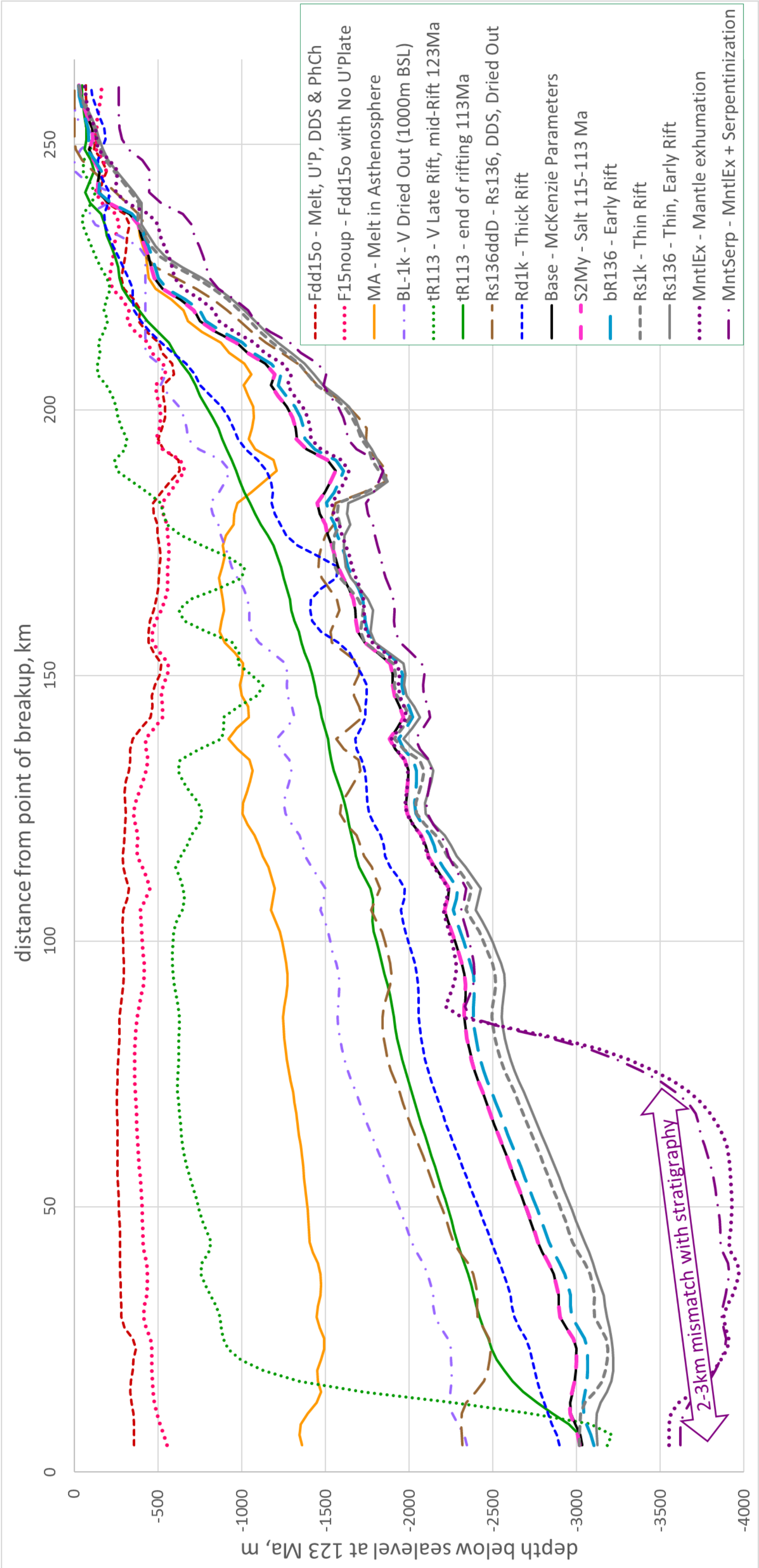

Figure S1. Depth of the syn-rift sediment surface at time of breakup, 123 Ma, for representative models in the accompanying article (Models Base, MA and Fdd15o, Fig.1a) plus additional models where the stratigraphic match is less than perfect.

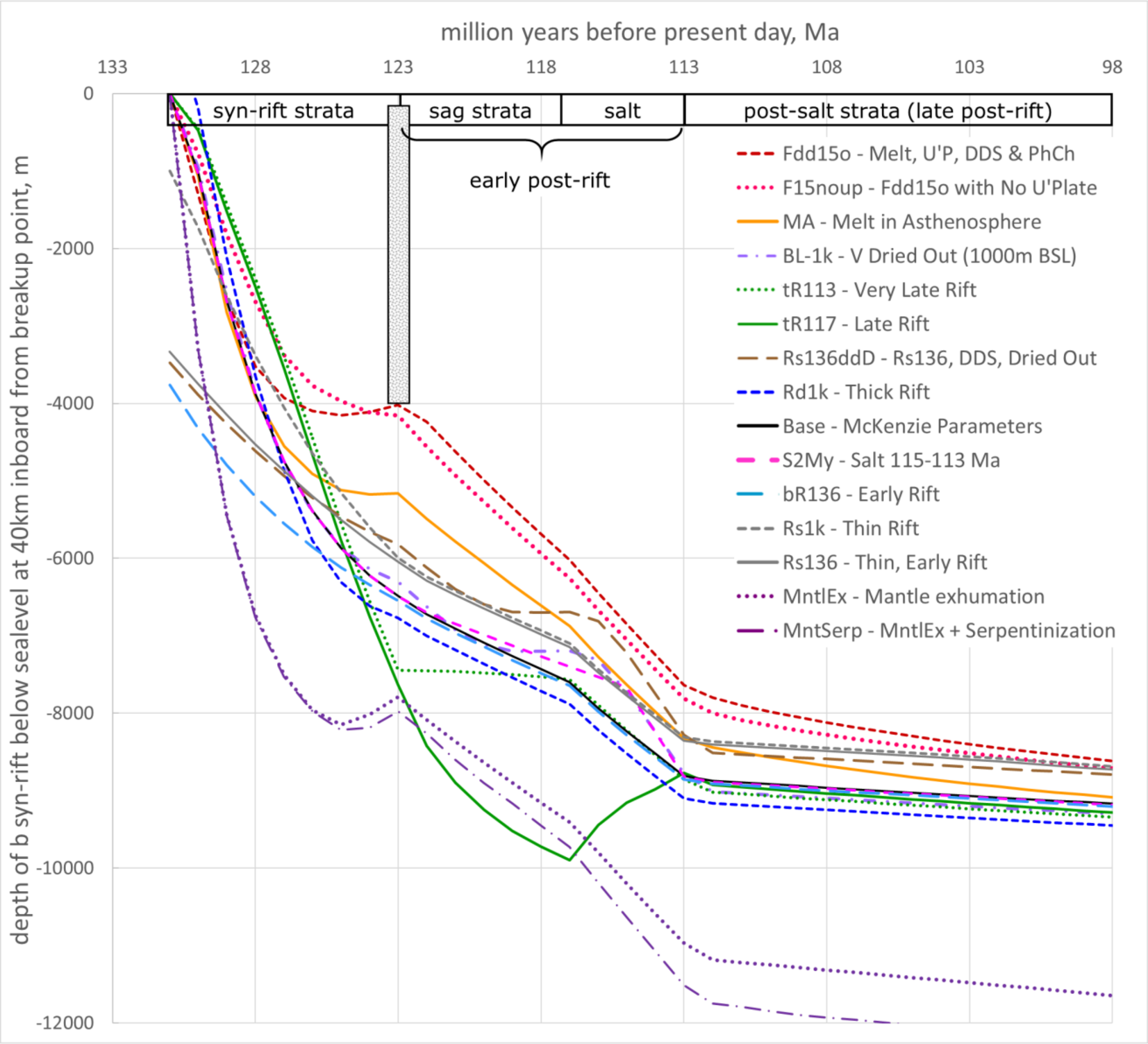

Figure S2. Graphs comparing subsidence histories of a 131 million year old surface (base of rift) for the models shown in Figure S1 at a position 40 km inboard of the breakup point. The grey bar indicates the thickness of syn-rift sediment at the time of breakup. Differences between the grey bar and a specific subsidence line at 123 Ma represents the water depth (unfilled accommodation space). Palaeobathymetric evidence of contemporaneous erosion and subsequent shallow water sag and salt deposits in the central South Atlantic region is contradicted where the water depth is significant (>500 m).

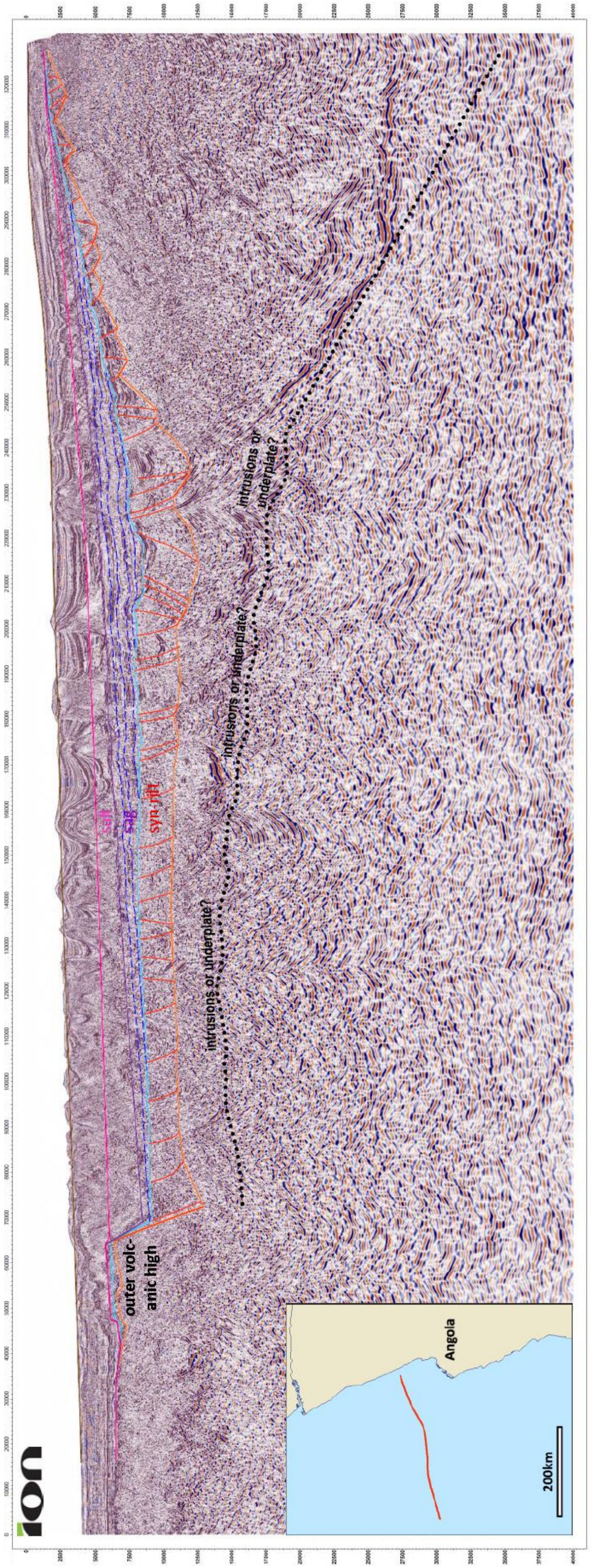

Figure S3. 2D pre-stack depth migrated seismic section from northern Kwanza Basin, offshore Angola, including the interpretation used as input for modeling of a non-volcanic rifted margin reported both here and in the accompanying article. Data is courtesy of ION GeoVentures (CongoSpan Seismic Line 2400). The field of view is 330 km (horizontal) by 40 km (vertical): the scales shown are in metres. West is on the left-hand (ocean) side of the section, east on the right (continent). Solid lines are the interpreted horizons used in TecMod, excluding indicative faults shown in red. Dashed dark blue lines indicate that flat layers within the sag progressively step (lap) onto the erosional surface at the top of the syn-rift interval (light blue line). The black dotted line indicates the base of crust which is an output from one of our final models (Fdd15o), its position correlating with the strong reflections generally interpreted to mark the Moho. The data was originally interpreted in Petrel 2016 and then exported as an image to Adobe Illustrator CC 2015 where the horizons were redrawn. The interpretation was then imported into TecMod as an SVG file to carry out the modeling. Brown horizon – seabed, 0 Ma; pink horizon – top salt (pre-salt diapir geometry), 113 Ma; purple horizon – base salt/top sag, 117 Ma; blue horizon – base sag/top syn-rift, 123 Ma; orange horizon – base syn-rift/top basement, 131 Ma. Oceanic crust starts at the outer volcanic high which we interpret to be a proto-oceanic ridge formed at the time of breakup, 123 Ma. The inset map shows the location of the section (red line). The same section is shown in Figs. 5 and 7 in Ref.[10] as well as others noted in the same reference.

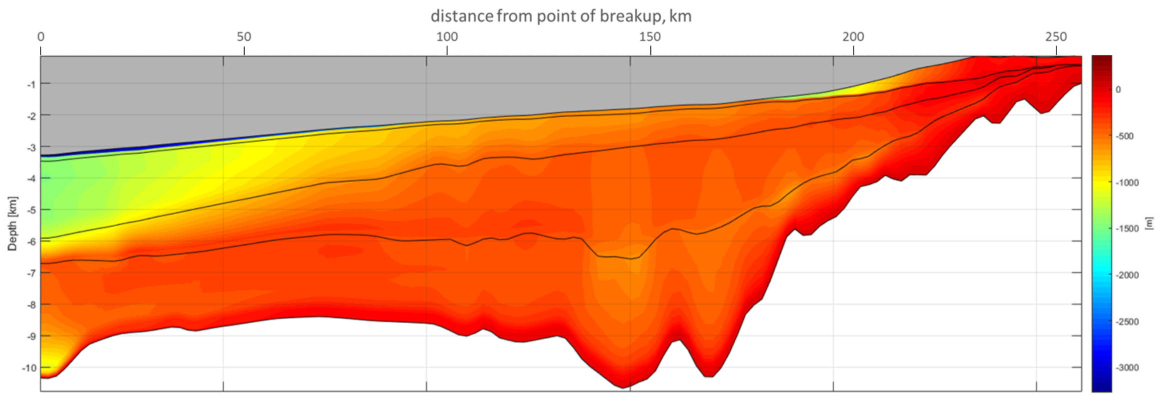

Fig. S4a

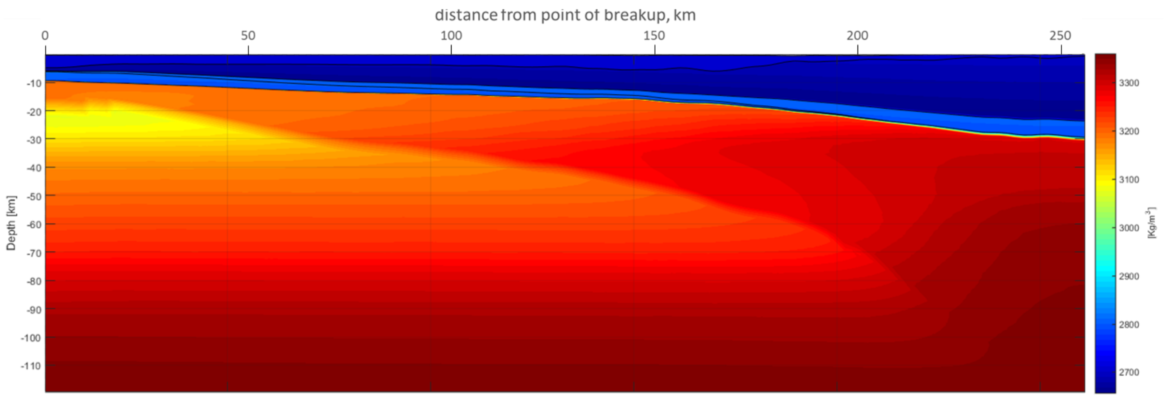

Fig. S4b

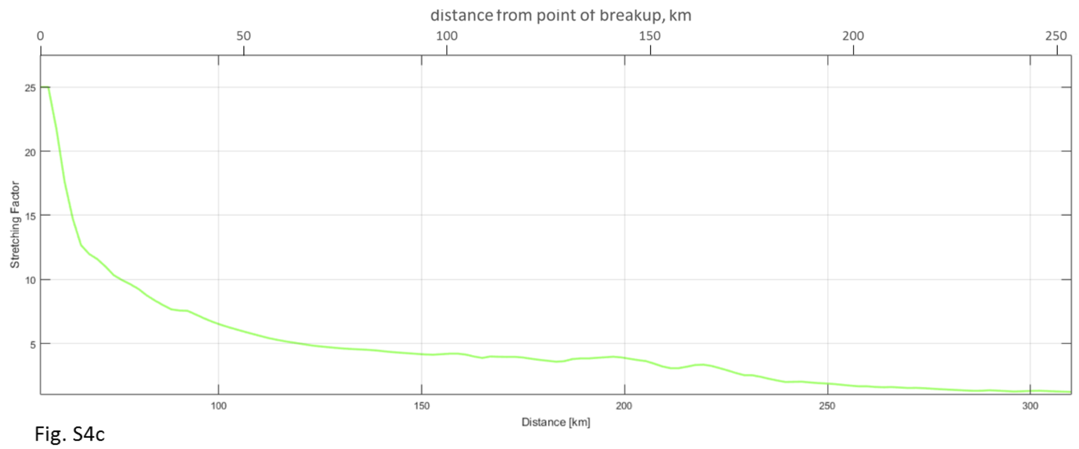

Fig. S4c

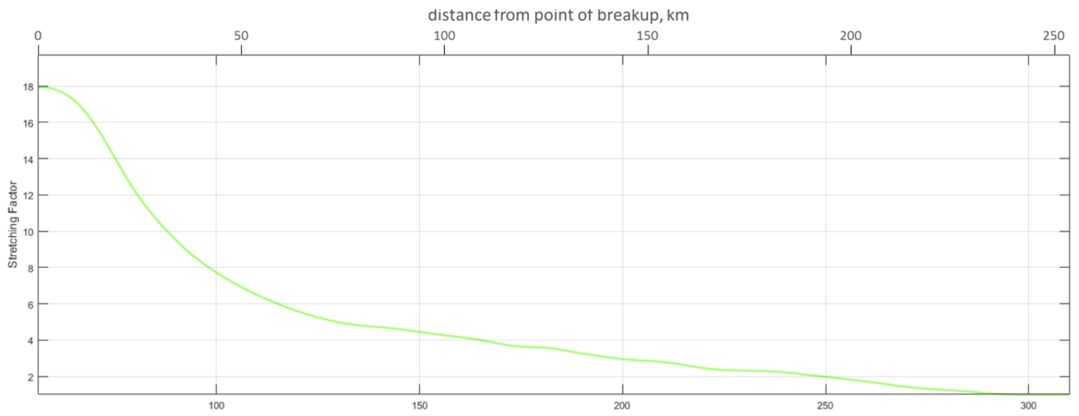

Fig. S4d

Figure S4. Cross-sections from TecMod model Fdd15o, supplemental to those shown in Fig.1 in the main article. **(a)** Model palaeobathymetry (depositional depth of sediments) shown in color. The scale is shown on the right, with water depth in metres. **(b)** Density of crust (blue colors) and density of asthenosphere (red-yellow colors) at the time of breakup. The scale is shown on the right, with densities in $kg.m^{-3}$. **(c)** Stretching factor for the crust (not including thickening by underplates). **(d)** Stretching factor for the mantle lithosphere.

Parameter	Description	Value	Units
	initial upper crustal thickness	28.0	km
	initial lower crustal thickness	7.0	km
	initial lithosphere thickness	120	km
	isostatic compensation depth	120	km
	initial height of land surface	500	m (above sea-level)
	basal lithosphere temperature	1603	^oK
	seafloor temperature	288	^oK
ρ	density		kg/m^3
c_i	specific heat capacity		$J/kg/^oK$
T	temperature		^oK
t	time		s
x	horizontal coordinate		m
z	vertical coordinate		m
v_x, v_z	horizontal and vertical velocity		m/s
A	radiogenic heat production in crust	2	$\mu W/m^3$
k	thermal conductivity		$W/m/^oK$
α	thermal expansion		$1/^oK$
ϕ	porosity		decimal value
c	inverse compaction		km^{-1}
c_0	parameter in pore fluid thermal conductivity	0.565	$W/m/^oK$
c_1	parameter in pore fluid thermal conductivity	$1.88E^{-03}$	$W/m/^oK^2$
c_2	parameter in pore fluid thermal conductivity	$-7.23E^{-06}$	$W/m/^oK^3$
c_f	parameter in grain thermal conductivity	250	^oK
D	flexural rigidity		$Pa.m^3$
g	gravitational acceleration		m/s^2
w	vertical deflection of the crust		m
S	kinematic deflection		m
q	differential load		kg/m^3
z	burial depth		m
	density of melt	2650	kg/m^3

Table S1. Model parameters.

Stratigraphic Name	Age Ma	Lithology	ϕ_i	c km^{-1}	α $1/^oK$	ρ kg/m^3	k $W/m/^oK$	c_i $J/Kg/^oK$	A W/m^3
	0								
post-salt		shale	0.62	0.50	0	2720	1.5	1000	1×10^{-6}
	113								
salt		halite	0.05	0	4.4×10^{-5}	2300	1.2	1000	1×10^{-6}
	117								
sag		90% sh, 10% lst	0.60	0.49	0	2710	1.5	1000	1×10^{-6}
	123								
syn-rift		50% ss, 50% clst	0.56	0.39	0	2705	2.6	1000	1×10^{-6}
	131								
upper crust		granite	0	0	2.4×10^{-5}	2700	2.5	1116	3×10^{-6}
lower crust		mafic	0	0	2.4×10^{-5}	2850	2.5	1116	2×10^{-6}
mantle lithosp		peridotite	0	0	3.2×10^{-5}	3300	3.4	1288	0
asthenosphere		fertile peridotite	0-0.16	0	3.2×10^{-5}	3300	3.4	1288	0

Table S2. Physical properties of rock types used in the models. Sh = shale; lst = limestone; ss = sandstone; clst = claystone.

Movie S1. 2D TecMod reconstruction for model Fdd15o through the upper part of the lithosphere showing the evolution of crust and sediment infill from 131 Ma to present day. Open with an internet browser. Colors indicate rock type. Pale blue = water.

Movie S2. 2D TecMod reconstruction for model Fdd15o through the lithosphere and upper part of the asthenosphere showing the melt fraction, indicated by colors, from 131 Ma to 91 Ma. Open with an internet browser.

Movie S3. 2D TecMod reconstruction for model Fdd15o through the lithosphere and upper part of the asthenosphere showing the density, indicated by colors, from 131 Ma to present day. Open with an internet browser.

Movie S4. Plate reconstruction for South Atlantic region based on work reported in Ref.[5]. Open with Microsoft PowerPoint, use Slide Show and the animation starts automatically on slide 2. The plate model was built using PaleoGIS software with the main assumptions shown on slides 15 and 16.

References

1. McKenzie, D. Some remarks on the development of sedimentary basins. *Earth and Planetary Science Letters* **40**, 25-32 (1978).
2. Dupré, S., Bertotti, G. & Cloetingh, S. Tectonic history along the South Gabon Basin: Anomalous early post-rift subsidence. *Marine and Petroleum Geology* **24**, 151-172 (2007).
3. Karner, G.D. & Gambôa, L.A.P. Timing and origin of the South Atlantic pre-salt sag basins and their capping evaporites. *Geological Society, London, Special Publications* **285**, 15-35 (2007).
4. Reston, T.J. The extension discrepancy and syn-rift subsidence deficit at rifted margins. *Petroleum Geoscience* **15**, 217-237 (2009).
5. Quirk, D.G. *et al.* Rifting, subsidence and continental break-up above a mantle plume in the central South Atlantic. *Geological Society, London, Special Publications* **369**, 185-214 (2013).
6. Esedo, R., van Wijk, J., Coblentz, D. & Meyer, R. Uplift prior to continental break-up: Indication for removal of mantle lithosphere? *Geosphere* **8**, 1078-1085 (2012).
7. Beglinger, S.E., Doust, H. & Cloetingh, S. Relating petroleum system and play development to basin evolution: West African South Atlantic basins. *Marine and Petroleum Geology* **30**, 1-25 (2012)
8. Chaboureau, A.-C. *et al.* Paleogeographic evolution of the central segment of the South Atlantic during Early Cretaceous times: Paleotopographic and geodynamic implications. *Tectonophysics* **604**, 191-223 (2013).
9. Quirk, D. Shakerley, A. & Howe, M.J. A mechanism for construction of volcanic rifted margins during continental breakup. *Geology* **42**, 1079-1082 (2014).
10. Cowie, L., Angelo, R.M., Kusznir, N., Manatschal, G. & Horn, B. Structure of the ocean-continent transition, location of the continent-ocean boundary and magmatic type of the northern Angolan margin from integrated quantative analysis of deep seismic reflection and gravity anomaly data. *Geological Society, London, Special Publications* **438**, 1-18 (2016).
11. Pindell, J., Graham, R. & Horn, B. Rapid outer marginal collapse at the rift to drift transition of passive margin evolution, with a Gulf of Mexico case study. *Basin Research* **26**, 1-25 (2014).
12. Crosby, A.G. *et al.* Evolution of deep-water rifted margins: Testing depth-dependent extensional models. *Tectonophysics* **30**, 1-36 (2004).
13. Huismans, R. & Beaumont, C. Depth-dependent extension, two-stage breakup and cratonic underplating at rifted margins. *Nature* **473**, 74-79 (2011)
14. Blaich, O.A. Faleide, J.I. & Tsikalas, F. Crustal breakup and continent-ocean transition at South Atlantic conjugate margins. *Journal of Geophysical Research* **116**, 1-38 (2011).
15. Péron-Pinvidic, G., Manatschal, G. & Osmundsen, P.T. Structural comparison of archetypal Atlantic rifted margins: A review of observations and concepts. *Marine and Petroleum Geology* **43**, 21-47 (2013).
16. Davison, I., Anderson, L. & Nuttall, P. Salt deposition, loading and gravity drainage in the Campos and Santos salt basins. Geological Society, London, Special Publications **363**, 159-173 (2012).
17. Braun, J. & Beaumont, C. A physical explanation of the relation between flank uplifts and the breakup unconformity at rifted continental margins. *Geology* **17**, 760-764 (1989).
18. Quirk, D.G. *et al.* Salt tectonics on passive margins: examples from Santos, Campos and Kwanza basins. *Geological Society, London, Special Publication* **363**, 207-244 (2012).

19. Hirsch, K.K., Bauer, K. & Scheck-Wenderoth, M. Deep structure of the western South African passive margin – Results of a combined approach of seismic, gravity and isostatic investigations. *Tectonophysics* **470**, 57-70 (2009).
20. Kusznir, N.J. & Karner, G.D. Continental lithospheric thinning and breakup in response to upwelling divergent mantle flow: application to the Woodlark, Newfoundland and Iberia margins. *Geological Society, London, Special Publication* **282**, 389-419 (2007).
21. Huismans, R.S. & Beaumont, C. Rifted continental margins: the case for depth-dependent extension. *Earth and Planetary Science Letters* **407**, 148-162 (2014).
22. White, R.S, Spence, G.D., Fowler, S.R., McKenzie, D.P., Westbrook, G.K. and Bowen, A.N., Magmatism at rifted continental margins. *Nature* **330**, 439-444 (1987).
23. Strozyk, F., Back, S. & Kukla, P.A. Comparison of the rift and post-rift architecture of conjugated salt and salt-free basins offshore Brazil and Angola/Namibia, South Atlantic. *Tectonophysics* **716**, 204-224 (2017).
24. Torsvik, T.H., Rousse, S., Labail, S.C. & Smethurst, M. A new scheme for opening of the South Atlantic Ocean and the dissection of an Aptian salt basin. *Geophysical Journal International* **177**, 1315-1333 (2009).
25. Aslanian, D. *et al.* Brazilian and African passive margins of the Central Segment of the South Atlantic Ocean: kinematic constraints. Tectonophysics **468**, 98-112 (2009).
26. Reston, T.J. The opening of the central segment of the South Atlantic: symmetry and the extension discrepancy. *Petroleum Geoscience* **16**, 199–206 (2010).
27. Péron-Pinvidic, G. *et al.* Unravelling the along-strike variability of the Angola-Gabon rifted margin: a mapping approach. *Geological Society, London, Special Publication* **438**, 1-28 (2015).
28. Moulin, M., Aslanian, D. & Unternehr, P. A new starting point for the South and Equatorial Atlantic Ocean. *Earth Science Reviews* **98**, 1-37 (2009).
29. Brune, S., Williams, S.E., Butterworth, N.P. & Müller, R.D. Abrupt plate accelerations shape rifted continental margins. *Nature* **536**, 201-204 (2016).
30. Müller, R.D. *et al.* Ocean basin evolution and global-scale plate reorganization events since Pangea breakup. *Annual Review of Earth and Planetary Sciences* **44**, 107-138 (2016).
31. Thiede, D. S. & Vasconcelos, P. M. Paraná flood basalts: Rapid extrusion hypothesis confirmed by new $^{40}Ar/^{39}Ar$ results. *Geology* **38**, 747-750 (2010).
32. Crosby, A.G. & McKenzie, D. An analysis of young ocean depth, gravity and global residual topography. *Geophysical Journal International* **178**, 1198-1219 (2009).
33. Al Hajri, Y., White, N. & Fishwick, S. Scales of transient convective support beneath Africa. *Geology* **37**, 883-886 (2009).
34. Winterbourne, J., Crosby, A. & White, N. Depth, age and dynamic topography of oceanic lithosphere beneath heavily sedimented Atlantic margins. *Earth and Planetary Science Letters* **287**, 137-151 (2009).
35. Goes S., Armitage, J., Harmon, N., Smith, H. & Huismans, R. Low seismic velocities below mid-ocean ridges: Attenuation versus melt retention. *Journal of Geophysical Research* **117**, 1-19 (2012).
36. Makris, J. & Ginzberg, A. The Afar Depression: transition between continental rifting and sea-floor spreading. *Tectonophysics* **141**, 199-214 (1987).
37. Tessema, A. & Antoine, L.A.G. Processing and interpretation of the gravity field of the East African Rift: implication for crustal extension. *Tectonophysics* **394**, 87-110 (2004).

38. Lewi, E. *et al.* Use of high-precision gravity survey to understand the formation of oceanic crust and the role of melt at the southern Red Sea rift in Afar, Ethiopia. *The Geological Society, London, Special Publications* **420**, 1-16 (2015).
39. Rychert, C.A. *et al.* Volcanism in the Afar Rift sustained by decompression melting with minimal plume influence. *Nature Geoscience* **5**, 1-4 (2012).
40. Mainprice, D. Modeling the anisotropic seismic properties of partially molten rocks found at mid-ocean ridges. *Tectonophysics* **279**, 161-179 (1997).
41. Harmon, N., Forsyth, D.W., Weeraratne, D.S., Yang, Y. & Webb, S.C. Mantle heterogeneity and off axis volcanism on young Pacific lithosphere. *Earth and Planetary Science Letters* **311**, 306-315 (2011).
42. Keranen, K.M., Klemperer, S.L., Julia, J., Lawrence, J.F. & Nyblade, A.A. Low lower crustal velocity across Ethiopia: Is the Main Ethiopian Rift a narrow rift in a hot craton? *Geochemistry Geophysics Geosystems* **10**, 1-19 (2009).
43. Jakovlev, A. *et al.* Seismic images of magmatic rifting beneath the western branch of the East African rift. *Geochemistry Geophysics Geosystems* **14**, 1-15 (2013).
44. Constable, S., Heinson, G.S., Anderson, G., & White, A. Seafloor electromagnetic measurements above axial seamount, Juan de Fuca Ridge. *Journal of Geomagnetism and Geoelectricity* **49**, 1327-1342 (1997).
45. Mechie, J., Keller, G.R., Prodehl, C., Khan, M.A. & Gaciri, S.J. A model for the structure, composition and evolution of the Kenya rift. *Tectonophysics* **278**, 95-119 (1997).
46. Sinha, M.C., Constable, S.C. & Peirce, C. Magmatic processes at slow spreading ridges: implications of the RAMESSES experiment at 57° 45´N on the Mid-Atlantic Ridge. *Geophysical Journal International* **135**, 731-745 (1998).
47. Constable, S. & Heinson, G., Hawaiian hot-spot swell structure from seafloor MT sounding. *Tectonophysics* **389**, 111-124 (2004).
48. Baba, K., Chave, A.D., Evans, R.L., Hirth, G. & Mackie, R.L. Mantle dynamics beneath the East Pacific Rise at 17°S: Insights from the Mantle Electromagnetic and Tomography (MELT) experiment. *Journal of Geophysical Research* **111**, 1-18 (2006).
49. Bastow, I.D., Nyblade, A.A., Stuart, G.W., Rooney, T.O. & Benoit, M.H. Upper mantle seismic structure beneath the Ethiopian hot spot: Rifting at the edge of the African low-velocity anomaly. *Geochemistry Geophysics Geosystems* **9**, 1-25 (2008).
50. Wang, Y., Forsyth, D.W. & Savage, B. Convective upwelling in the mantle beneath the Gulf of California. *Nature* **462**, 499-502 (2009).
51. Stork, A.L., Stuart, G.W., Henderson, C.M., Keir, D. & Hammond, J.O.S. Uppermost mantle (P_n) velocity model for the Afar region, Ethiopia: an insight into rifting processes. *Geophysical Journal International* **193**, 1-8 (2013).
52. Birt, C.S. *et al.* The influence of pre-existing structures on the evolution of the southern Kenya Rift Valley – Evidence from seismic and gravity studies. *Tectonophysics* **278**, 211-242 (1997).
53. Evans, R.L. *et al.* Compositional controls on oceanic plates: geophysical evidence from the MELT area. *Nature* **437**, 249-252 (2005).
54. Heinson, G., Constable, S. & White, A. Episodic melt transport at mid-ocean ridges inferred from magnetotelluric sounding. *Geophysical Research Letters* **27**, 2317-2320 (2000).
55. Hammond, W.C. & Toomey, D.R. Seismic velocity anisotropy and heterogeneity beneath the Mantle Electromagnetic and Tomography Experiment (MELT) region of the East Pacific Rise from analysis of *P* and *S* body waves. *Journal of Geophysical Research* **108**, 1-24 (2003).

56. Yang, Y., Forsyth, D.W. & Weeraratne, D.S. Seismic attenuation near the East Pacific Rise and the origin of the low-velocity zone. *Earth and Planetary Science Letters* **258**, 260-268 (2007).
57. Hammond, J.O.S. *et al.* The nature of the crust beneath the Afar triple junction: evidence from receiver functions. *Geochemistry Geophysics Geosystems* **12**, 1-24 (2011).
58. Hammond, J.O.S. *et al.* Mantle upwelling and initiation of rift segmentation beneath the Afar Depression. *Geology* **41**, 635-638 (2013).
59. Rychert, C.A. Laske, G., Harmon, N. & Shearer, P.M. Seismic imaging of melt in a displaced Hawaiian plume. *Nature Geoscience* **6**, 657-660 (2012).
60. Gallacher, R.J. *et al.* The initiation of segmented buoyancy-driven melting during continental breakup. *Nature Communications* **7** 1-9 (2016).
61. Hernlund, J.W., Tackley, P.J. & Stevenson, D.J. Buoyant melting instabilities beneath extending lithosphere: 1. Numerical models. *Journal of Geophysical Research* **113**, 1-17 (2008).
62. McKenzie, D.P. The compaction of igneous and sedimentary rocks. *The Journal of the Geological Society, London* **144**, 299-307 (1987).
63. Schutt, D.L. & Lesher, C.E. Effects of melt depletion on the density and seismic velocity of garnet and spinel lherzolite. *Journal of Geophysical Research* **111**, 1-24 (2006).
64. Ferguson, D.J. *et al.* Melting during late-stage rifting in Afar is hot and deep. *Nature* **499**, 70-73 (2013).
65. Lee, C.-T.,A., Luffi, P., Plank, T., Dalton, H. & Leeman, W.P. Constraints on the depths and temperatures of basaltic magma generation on Earth and other terrestrial planets using new thermobarometers for mafic magmas. *Earth and Planetary Science Letters* **279**, 20-23 (2009).
66. Foucher, J.-P., Le Pichon, X. & Sibuet, J.-C. The ocean-continent transition in the uniform lithospheric stretching model: role of partial melting in the mantle. *Philosophical Transactions of the Royal Society A* **305**, 541-557 (1981).
67. Faul, U.H. Melt retention and segregation beneath mid-ocean ridges *Nature* **410**, 920-923 (2001).
68. Rooney, T.O., Herzberg, C & Bastow, I.D. Elevated mantle temperature beneath East Africa. *Geology* **40**, 27-30 (2012).
69. McKenzie, D.P. & Bickle, M.J. The volume and composition of melt generated by extension of the lithosphere. *Journal of Petrology* **29**, 625-679 (1988).
70. Chantel, J. *et al.* Experimental evidence supports mantle partial melting in the asthenosphere. *Science Advances* **2**, 1-7 (2016).
71. Hirschmann, M.M. Mantle solidus: experimental constraints and the effect of peridotite composition. *Geochemistry Geophysics Geosystems* **1**, 1-26 (2000).
72. Katz, R.F., Spiegelman, M & Langmuir, C.H. A new parameterization of hydrous mantle melting. *Geochemistry Geophysics Geosystems* **4**, 1-19 (2003).
73. Bown, J.W. & White, R.S. Effect of finite extension rate on melt generation at rifted continental margins. *Journal of Geophysical Research* **100**, 18.011-18.029 (1995).
74. Schmeling, H. Numerical models on the influence of partial melt on elastic, anelastic and electric properties of rocks. Part I: elasticity and anelasticity. *Physics of the Earth and Planetary Interiors* **41**, 34-57 (1985).
75. Utada, H. & Baba, K. Estimating the electrical conductivity of the melt phase of a partially molten asthenosphere from seafloor magnetotelluric sounding data. *Physics of the Earth and Planetary Interiors* **227**, 41-47 (2014).

76. Hier-Majumder, S. & Drombosky, T. Development of anisotropic contiguity in deforming partially molten aggregates: 2. Implications for the lithosphere-asthenosphere boundary. *Journal of Geophysical Research: Solid Earth* **120**, 1-14 (2015).
77. Hammond, J.O.S. & Kendall, J.-M. Constraints on melt distribution from seismology: a case study in Ethiopia. *Geological Society, London, Special Publication* **420**, 1-21 (2016).
78. Cheadle, M.J., Elliott, M.T. & McKenzie, D. Percolation threshold and permeability of crystallizing igneous rocks: The importance of textural equilibrium. *Geology* **32**, 757-760 (2004).
79. Yoshino, T., Yamazaki, D. & Mibe, K. Well-wetted olivine grain boundaries in partially molten peridotite in the asthenosphere. *Earth and Planetary Science Letters* **283**, 167-173 (2009).
80. Maumus, J., Bagdassarov, N. & Schmeling, H. Electrical conductivity and partial melting of mafic rocks under pressure. *Geochimica et Cosmochimica Acta* **69**, 4703-4718 (2005).
81. Pervukhina, M. & Kuwahara, Y. Correlations between electrical and elastic properties of solid–liquid composites with interfacial energy-controlled equilibrium microstructures. *Earth and Planetary Science Letters* **265**, 410–422 (2008).
82. White, R. & McKenzie, D. Magmatism at rift zones: the generation of volcanic continental margins and flood basalts. *Journal of Geophysical Research* **94**, 7685-7729 (1989).
83. Armitage, J.J. *et al.* Upper mantle temperature and the onset of extension and break-up in Afar, Africa, *Earth and Planetary Science Letters* **418**, 78-90 (2015).
84. Wallner, H. & Schmeling, H. Numerical models of mantle lithosphere weakening, erosion and delamination induced by melt extraction and emplacement. *International Journal of Earth Sciences (Geol Rundsch)* **105**, 1741-1760 (2016).
85. Forsyth, D.W. Geophysical constraints of mantle flow and melt generation beneath mid-ocean ridges. *Mantle Flow and Melt Generation at Mid-Ocean Ridges, Geophysical Monograph 71, American Geophysical Union, Washington DC.,* 1-65 (1992).
86. McKenzie, D. The extraction of magma from the crust and mantle. *Earth and Planetary Science Letters* **74**, 81-91 (1985).
87. Nielsen, T.K. & Hopper, J.R. From rift to drift: Mantle melting during continental breakup. *Geochemistry Geophysics Geosystems* **5**, 1-24 (2004).
88. Dunn, R.A. & Forsyth, D.W. Imaging the transition between the region of mantle melt generation and the crustal magma chamber beneath the southern East Pacific Rise with short-period Love waves. *Journal of Geophysical Research* **108** 1-20 (2003).
89. Hier-Majumder, S., Ricard, Y. & Bercovici, D. Role of grain boundaries in magma migration and storage. *Earth and Planetary Science* Letters **248**, 735-749 (2006).
90. Toramaru, A. & Fujii, N. Connectivity of melt phase in a partially molten peridotite. *Journal of Geophysical Research* **91**, 9239-9252 (1986).
91. Key, K., Constable, S., Liu, L. & Pommier, A. Electrical image of passive mantle upwelling beneath the northern East Pacific Rise. *Nature* **495**, 499-503 (2013).
92. Desissa, M. *et al.* A mantle magma reservoir beneath an incipient mid-ocean ridge in Afar, Ethiopia. *Nature Geoscience* **6**, 861-865 (2013).
93. Takei, Y. & Hier-Majumder, S. A generalized formulation of interfacial tension driven fluid migration with dissolution/precipitation. *Earth and Planetary Science Letters* **288**, 138-148 (2009).
94. Tirone, M., Sen, G. & Morgan J.P. Petrological geodynamic modeling of mid-ocean ridges. *Physics of the Earth and Planetary Interiors* **190-191**, 51-70 (2012).

95. Wölbern, I., & Rümpke, G. Melt infiltration of the lower lithosphere beneath the Tanzania craton and the Albertine rift inferred from S receiver functions. *Geochemistry Geophysics Geosystems* **13**, 1-20 (2012).
96. Korenaga, J., Kelemen, P.B. & Holbrook, W.S. Methods for resolving the origin of large igneous provinces from crustal seismology. *Journal of Geophysical Research* **107**, 1-27 (2002).
97. White, R.S. *et al.* Lower-crustal intrusion on the North Atlantic continental margin. *Nature*, **452**, 460-465 (2008).
98. Plank, T. & Forsyth, D.W. Thermal structure and melting conditions in the mantle beneath the Basin and Range province from seismology and petrology. *Geochemistry Geophysics Geosystems* **17**, 1312-1338 (2016).
99. Scott, D.R. & Stevenson, D.J. A self-consistent model of melting, magma migration and buoyancy-driven circulation beneath mid-ocean ridges. *Journal of Geophysical Research* **94**, 2973-2988 (1989).
100. Eilon, Z.C. & Abers, G.A. High seismic attenuation at a mid-ocean ridge reveals the distribution of deep melt. *Science Advances* **3**, 1-8 (2017).
101. Gallacher, R.J. *et al.* The initiation of segmented buoyancy-driven melting during continental breakup. *Nature Communications* **7** 1-9 (2016).
102. Courtillot, V., Jaupart, C., Manighetti, I., Tapponnier, P. & Bess, J. On causal links between flood basalts and continental breakup. *Earth and Planetary Science Letters* **166**, 177-195 (1999).
103. Bronner, A., Sauter, D., Manatschal, G., Péron-Pinvidic, G. & Munschy M. Magmatic breakup as an explanation for magnetic anomalies at magma-poor rifted margins. *Nature Geoscience* **4**, 549-553 (2011).
104. Storey, M., Duncan, R.A. & Tegner, C. Timing and duration of volcanism in the North Atlantic Igneous Province: Implications for geodynamics and links to the Iceland hotspot. *Chemical Geology* **241**, 264-281 (2007).
105. Hebert, L.B. & Montési, L.G.J. Melt extraction pathways at segmented oceanic ridges: application to the East Pacific Rise at the Siqueiros transform. *Geophysical Research Letters* **38**, L11306, 1-5 (2011).
106. Rüpke, L.H., Schmid, D.W. Hartz, E.H. & Martinsen, B. Basin modeling of a transform margin setting: structural, thermal and hydrocarbon evolution of the Tano Basin, Ghana. *Petroleum Geoscience* **16**, 283-298 (2010).
107. Rüpke, L.H., Schmalzholz, S.M., Schmid, D.W. & Podladchikov, Y.Y. Automated reconstruction of sedimentary basins using two-dimensional thermo-tectono-stratigraphic forward models – tested on the Northern Viking Graben. *AAPG Bulletin* **92**, 309-326 (2008).
108. Nocedal, J. & Wright, S.J. Numerical Optimization. *Springer: New York* (1999).
109. Fletcher, R., Practical Methods of Optimization. *John Wiley and Sons: Chichester* (2000).
110. White, N. & Bellingham, P. A two-dimensional inverse model for extensional sedimentary basins 1. Theory. *Journal of Geophysical Research* **107**, ETG17-1–ETG17-20 (2008).
111. Poplavskii, K.N., Podladchikov, Y.Y. & Stephenson, R.A. Two-dimensional inverse modeling of sedimentary basin subsidence. *Journal of Geophysical Research* **106**, 6657-6671 (2001).
112. Woodside, W. & Woodside, J.H. Thermal conductivity of porous media: I. Unconsolidated Sands. *Journal of Applied Physics* **32**, 1688-1699 (1961).
113. Deming, D. & Chapman, D.S. Thermal histories and hydrocarbon generation – example from Utah-Wyoming thrust belt. *AAPG Bulletin* **73**, 1455-1471 (1989).

114. Beardsmore, G.R. & Cull, J.R. Crustal Heat Flow. *Cambridge University Press: Cambridge* (2001).
115. Kaus, B.J.P., Connolly, J.A.D., Podladchikov, Y.Y. & Schmalholz, S.M. Effect of mineral phase transitions on sedimentary basin subsidence and uplift. *Earth and Planetary Letters* **233**, 213-228 (2005).
116. Simon, N.S.C. & Podladchikov, Y.Y. The effect of mantle composition on density in the extending lithosphere. *Earth and Planetary Science Letters* **272**, 148-157 (2008).
117. Weissel, J.K. & Karner, G.D. Flexural uplift of rift flanks due to mechanical uploading of the lithosphere during extension. *Journal of Geophysical Research – Solid Earth and Planets* **94**, 13919-13950 (1989)
118. Kooi, H., Cloetingh, S. & Burrus, J. Lithospheric necking and regional isostasy at extensional basins. I. Subsidence and gravity modeling with an application to the Gulf of Lion Margin (SE France). *Journal of Geophysical Research* **97** 17553-17571 (1992).
119. Kusznir, N.J. & Ziegler, P.A. The mechanics of continental extension and sedimentary basin formation – a simple-shear pure-shear flexural cantilever model. *Tectonophysics* **215**, 117-131 (1992).
120. Watts, A.B., Karner, G.D & Steckler, M.S. Lithospheric flexure and the evolution of sedimentary basins. *The Philosophical Transactions of the Royal Society of London, Series A – Mathematical, Physical and Engineering Sciences* **305**, 249-281 (1982).
121. Debremaecker, J.C. Temperature, subsidence and hydrocarbon maturation in extensional basins – a finite-element model. *AAPG Bulletin* **67**, 1410-1414 (1983).
122. Lucazeau, F. & Ledouaran, S. The blanketing effect of sediments in basins formed by extension – a numerical-model – application to the Gulf of Lion and Viking Graben. *Earth and Planetary Science Letters* **74**, 92-102 (1985).
123. Wangen, M. The blanketing effect in sedimentary basins. *Basin Research* **7**, 283-298 (1995)
124. Theissen, S. & Rüpke, L.H. Feedbacks of sedimentation on crustal heat flow: new insights from the Voring Basin, Norwegian Sea. **22**, 976-990 (2010).
125. Royden, L. & Keen, C.E. Rifting process and thermal evolution of the continental margin of eastern Canada determined from subsidence curves. *Earth and Planetary Science Letters* **51**, 343-361 (1980).
126. Sclater, J.G. & Christie, P.A.F. Continental stretching – explanation of post-Cretaceous subsidence of Central North Sea Basin. *AAPG Bulletin* **64**, 781-782 (1980).
