# Supplementary figures and images for: Melt-induced buoyancy may explain the elevated rift-rapid sag paradox during breakup of continental plates
Source: Sci Rep. 2018 Jul 3;8:9985. doi: 10.1038/s41598-018-27981-2 (PMC6030112; doi:10.1038/s41598-018-27981-2)

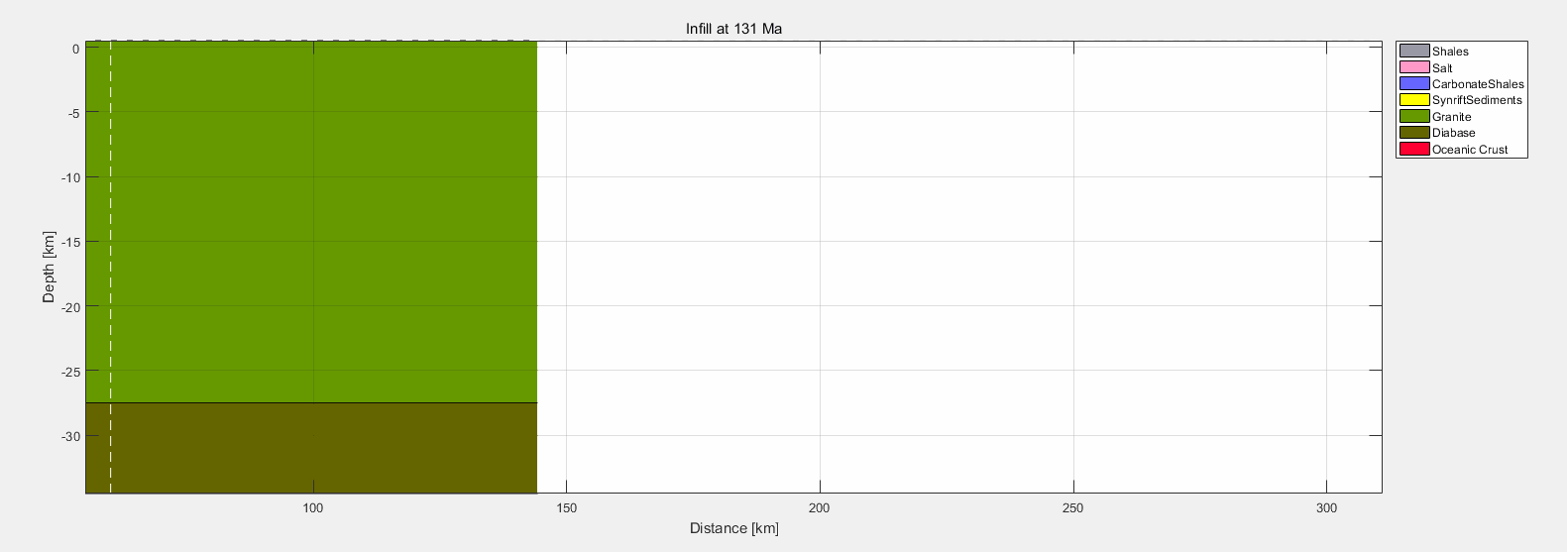

Supplement: Supplementary file 2 — Supplementary Movie S1 [file 41598_2018_27981_MOESM2_ESM.gif]

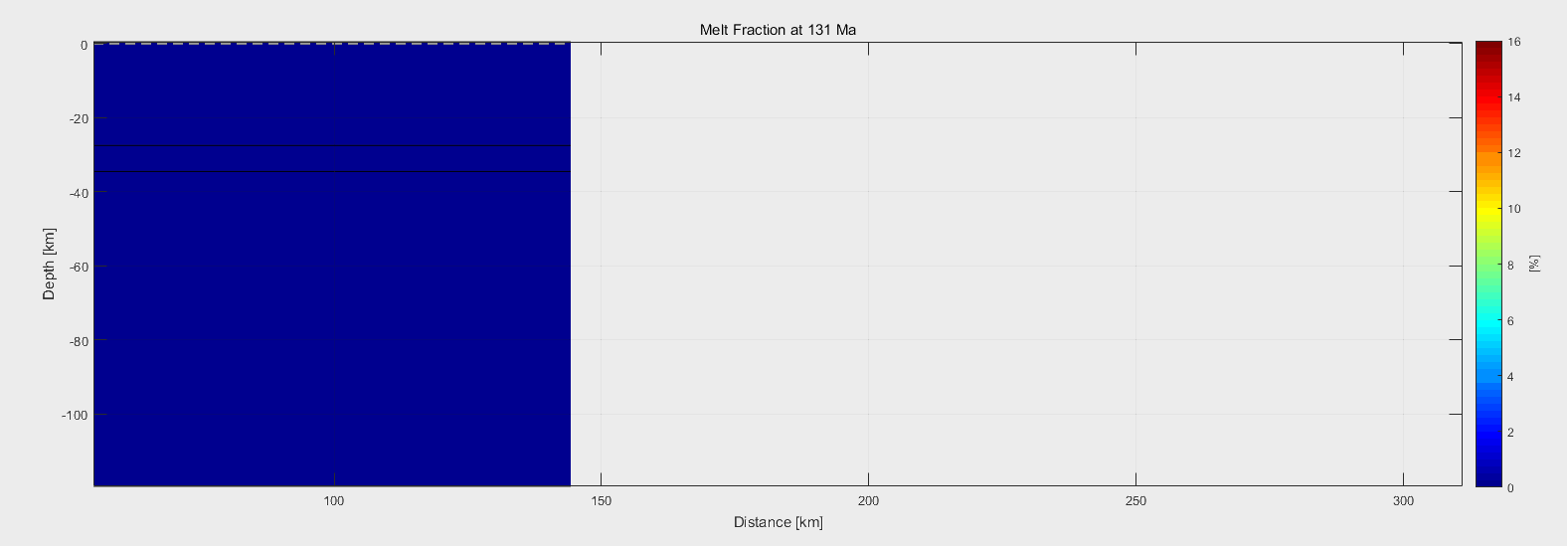

Supplement: Supplementary file 3 — Supplementary Movie S2 [file 41598_2018_27981_MOESM3_ESM.gif]

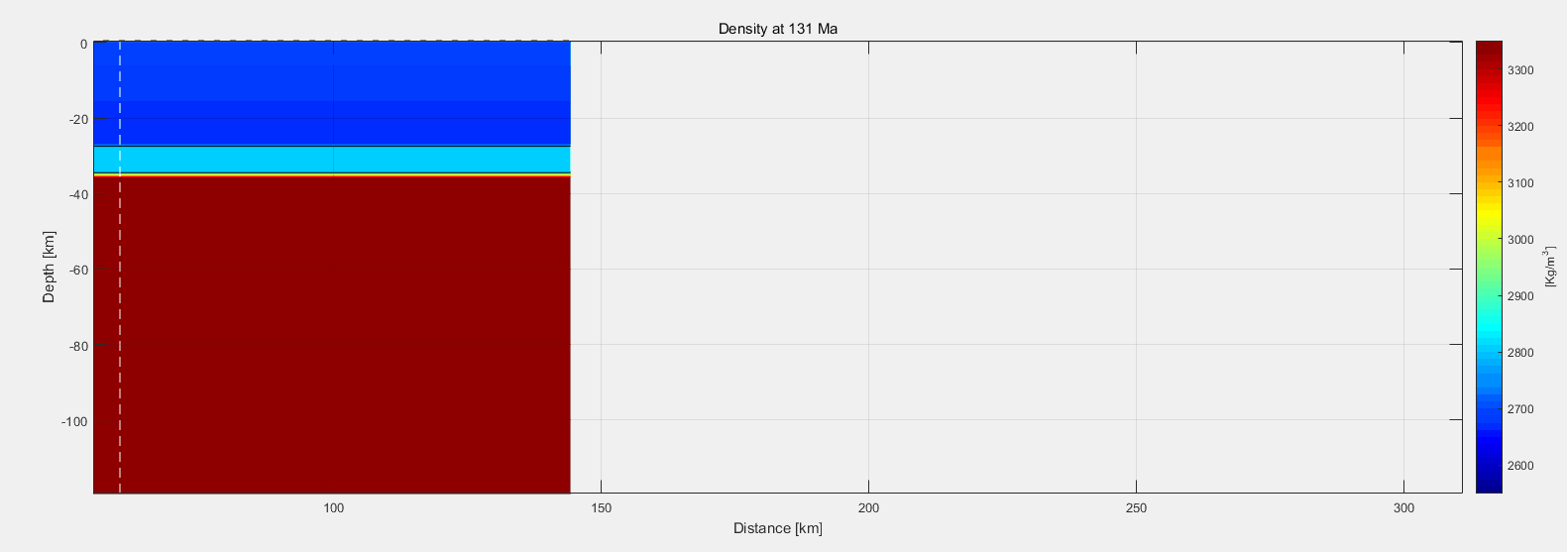

Supplement: Supplementary file 4 — Supplementary Movie S3 [file 41598_2018_27981_MOESM4_ESM.gif]
