# Supplementary material for: Melt-induced buoyancy may explain the elevated rift-rapid sag paradox during breakup of continental plates
Source: Sci Rep. 2018 Jul 3;8:9985. doi: 10.1038/s41598-018-27981-2 (PMC6030112; doi:10.1038/s41598-018-27981-2)
Supplement: Supplementary file 5 — Supplementary Movie S4, plate reconstruction [file 41598_2018_27981_MOESM5_ESM.ppt]

## Slide 1
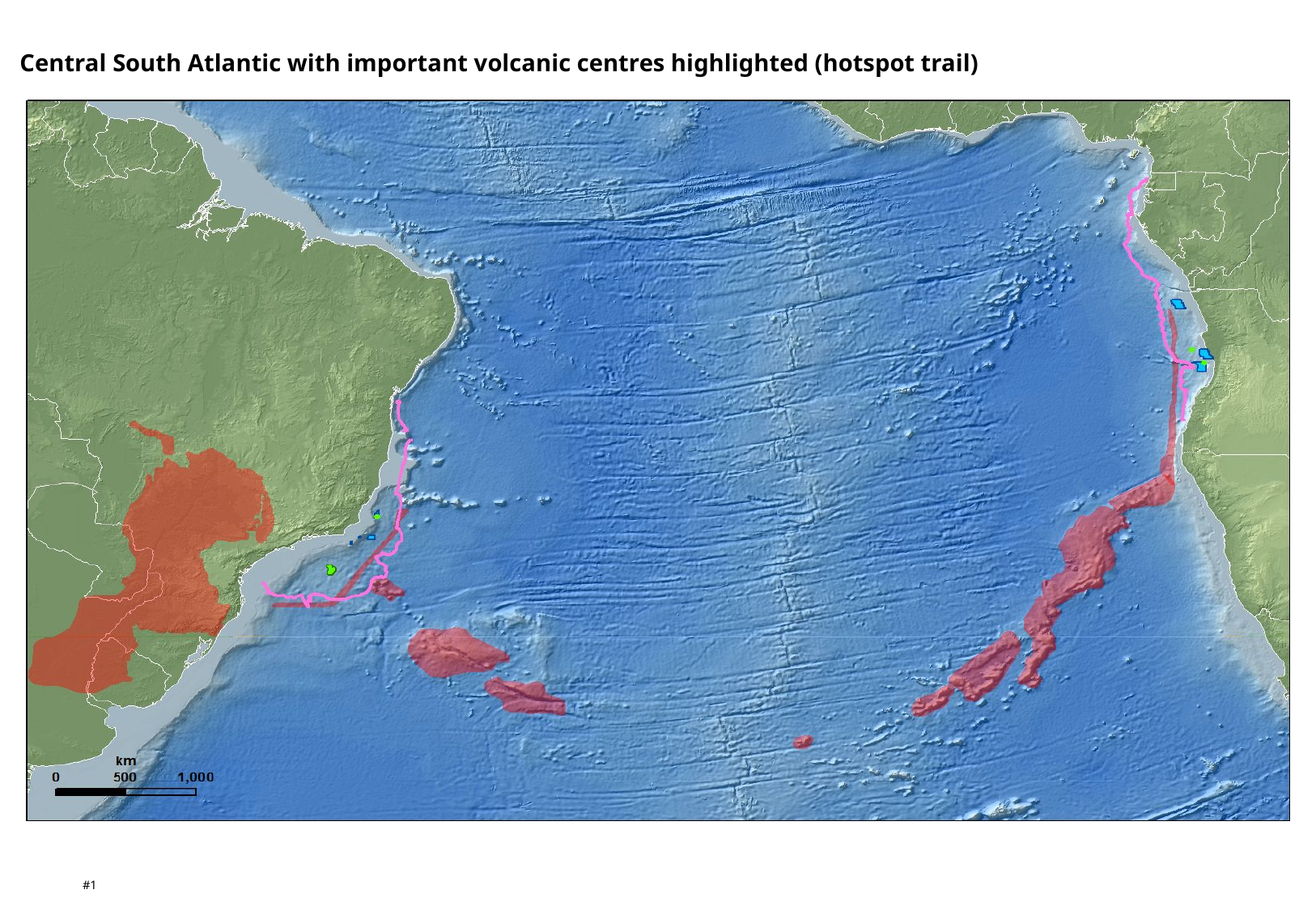

Central South Atlantic with important volcanic centres highlighted (hotspot trail)
#<number>

## Slide 2
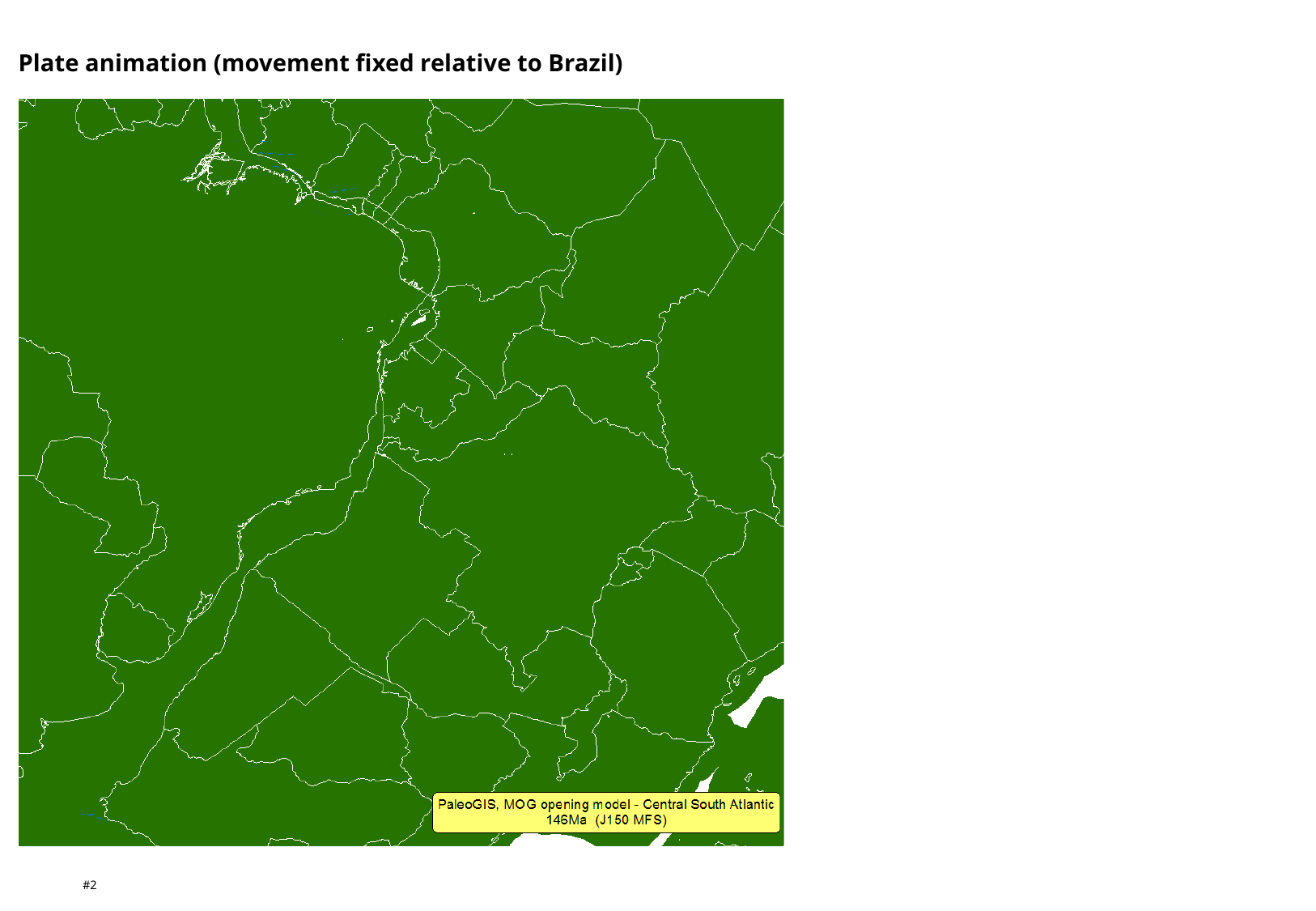

Plate animation (movement fixed relative to Brazil)
#<number>

## Slide 3
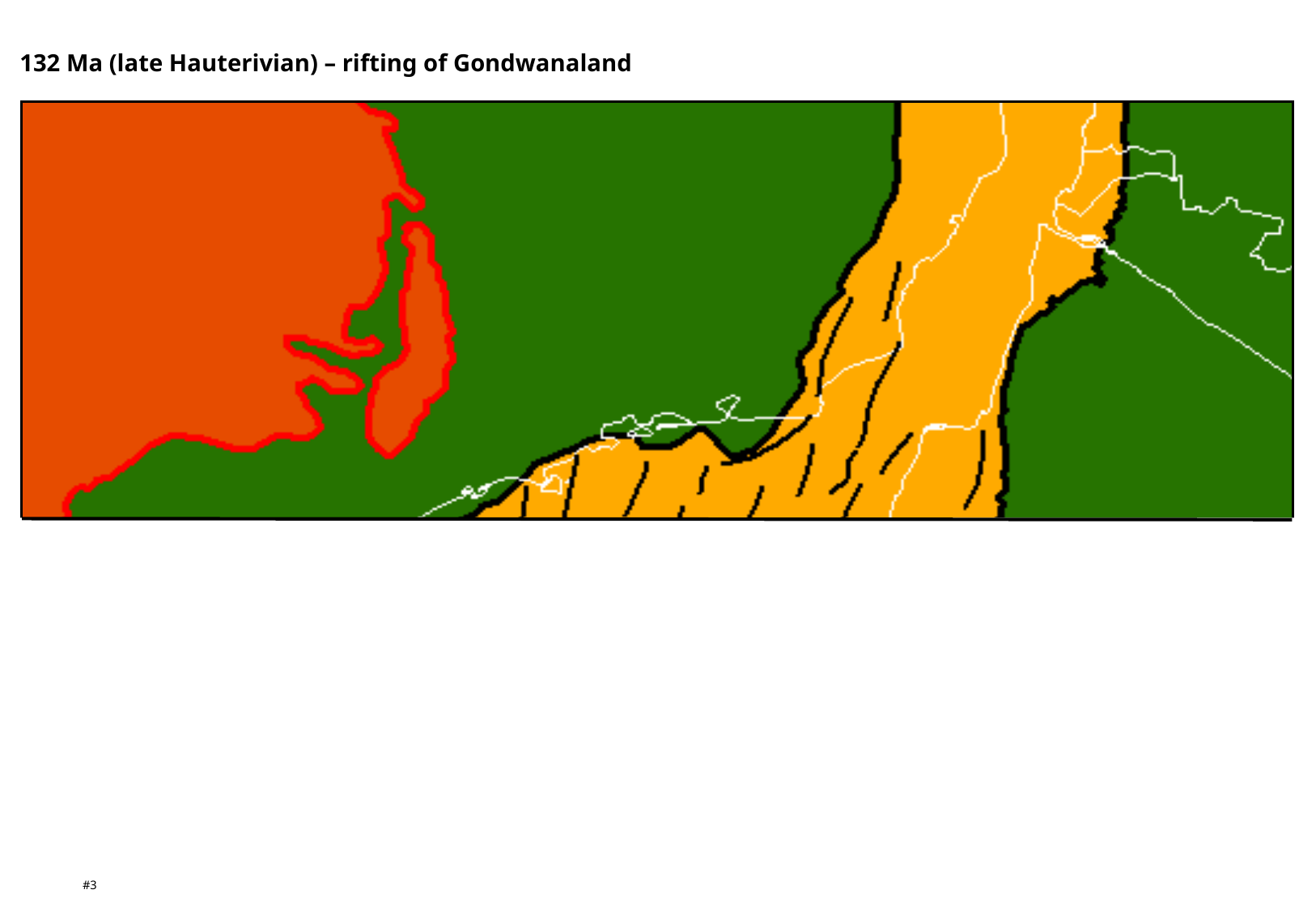

132 Ma (late Hauterivian) – rifting of Gondwanaland
#<number>

## Slide 4
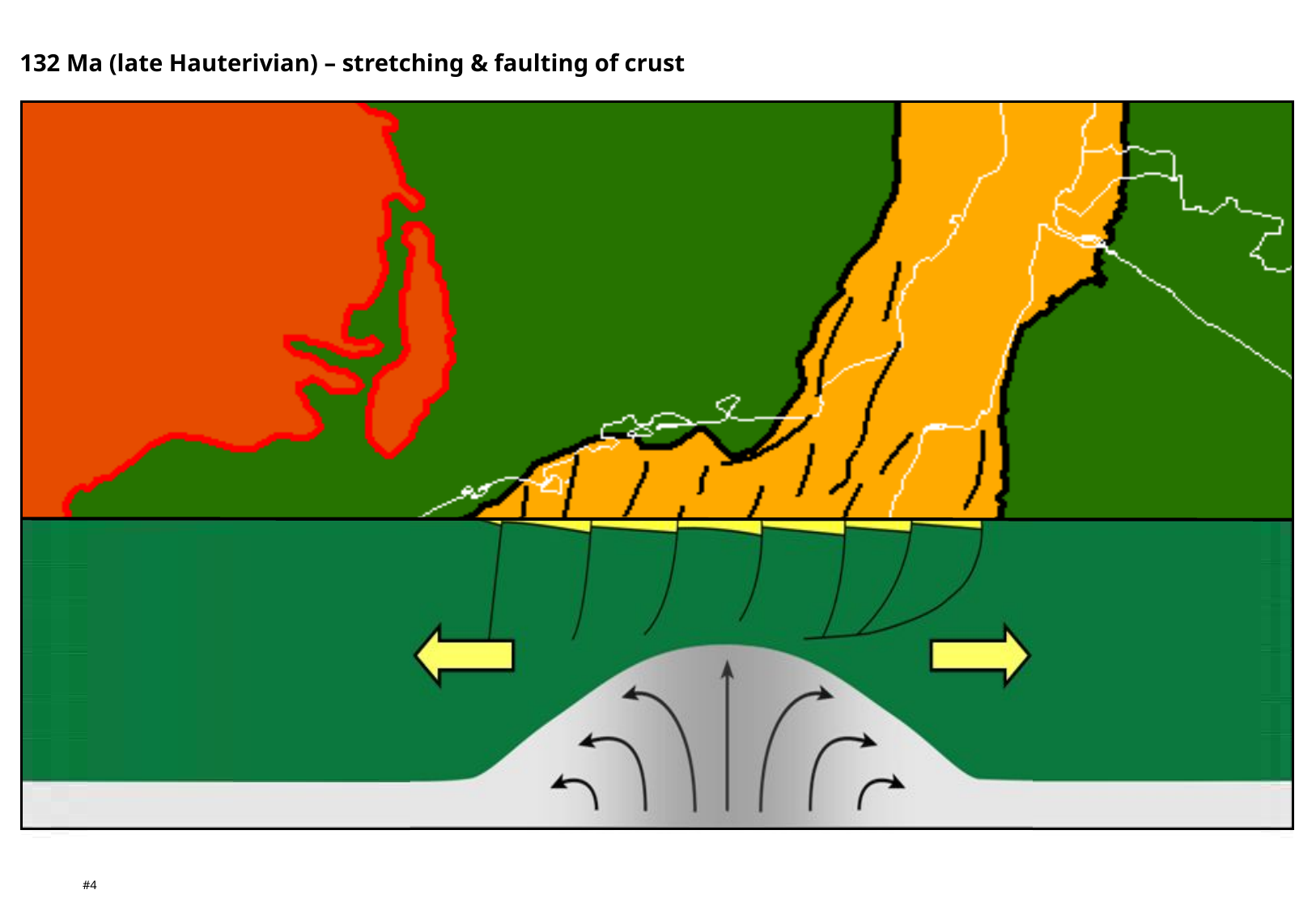

132 Ma (late Hauterivian) – stretching & faulting of crust
#<number>

## Slide 5
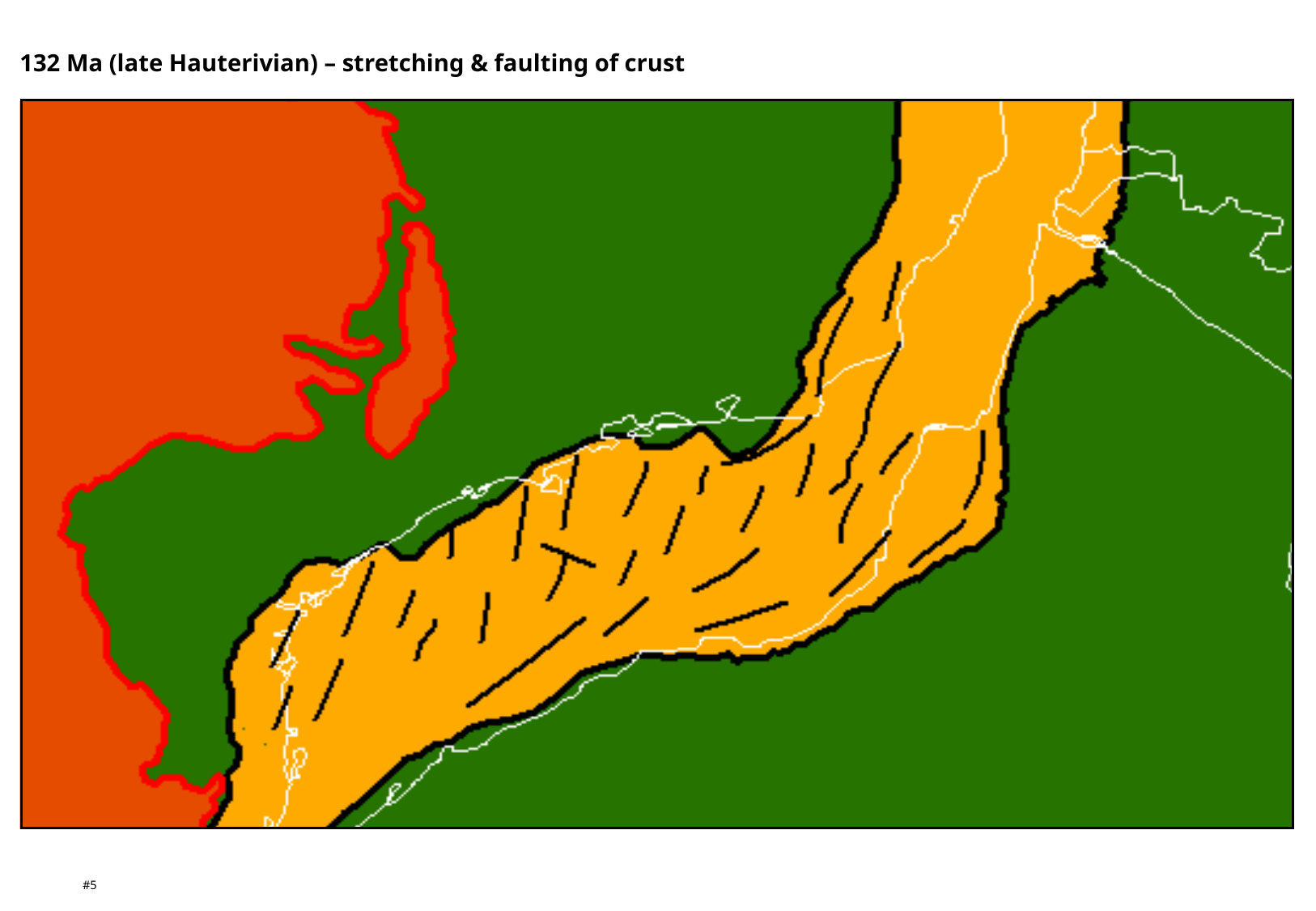

132 Ma (late Hauterivian) – stretching & faulting of crust
#<number>

## Slide 6
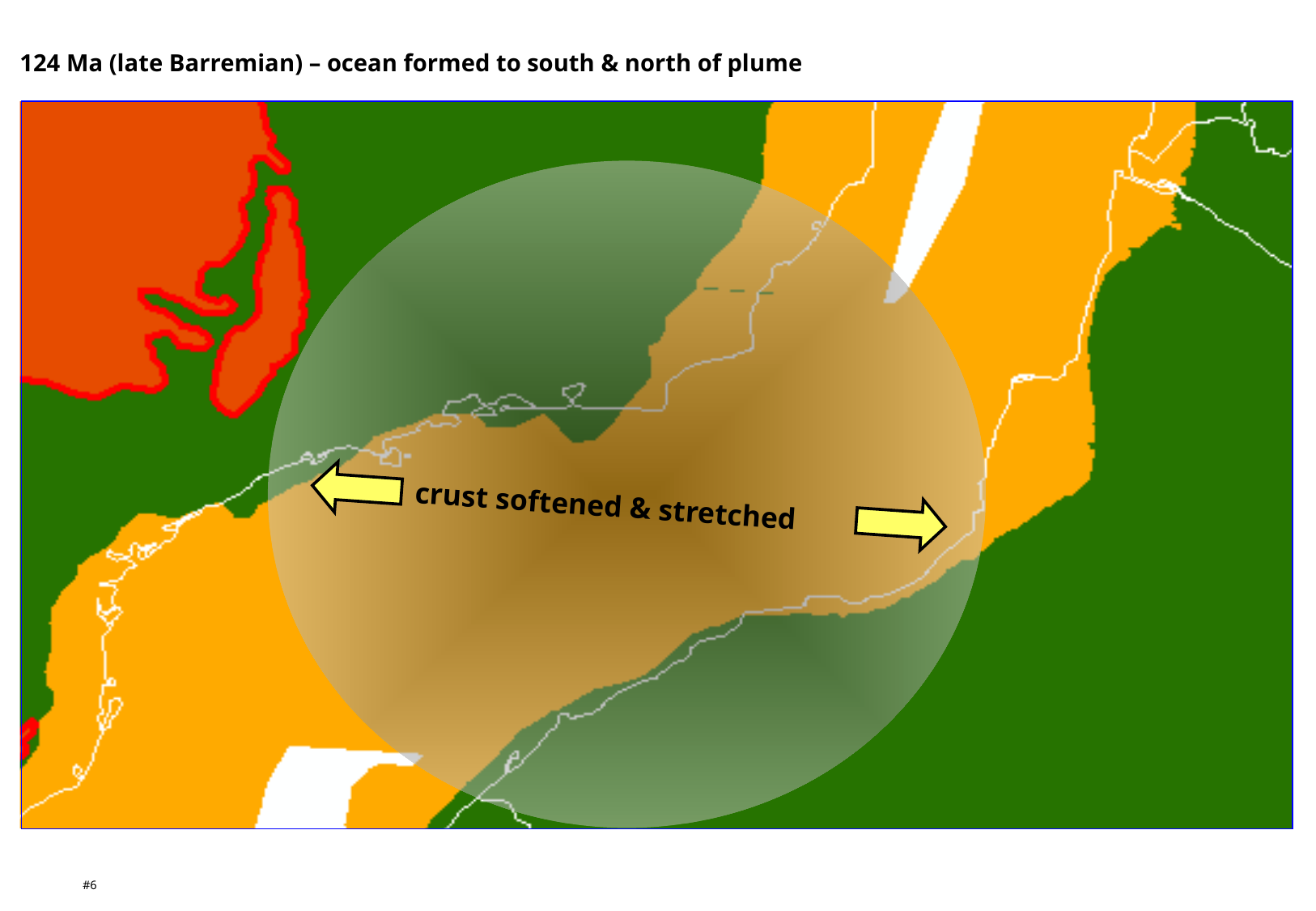

124 Ma (late Barremian) – ocean formed to south & north of plume
crust softened & stretched
#<number>

## Slide 7
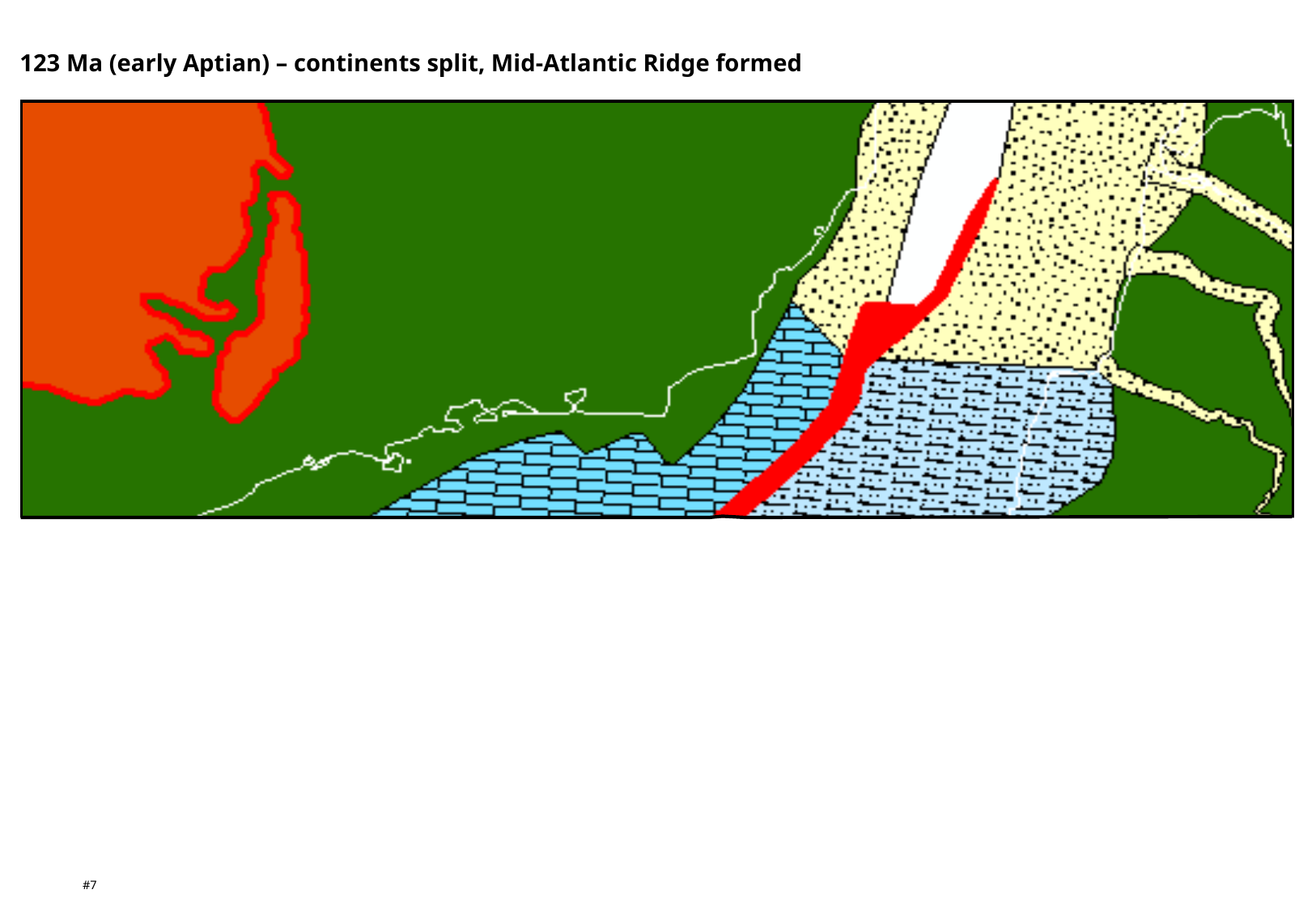

123 Ma (early Aptian) – continents split, Mid-Atlantic Ridge formed
#<number>

## Slide 8
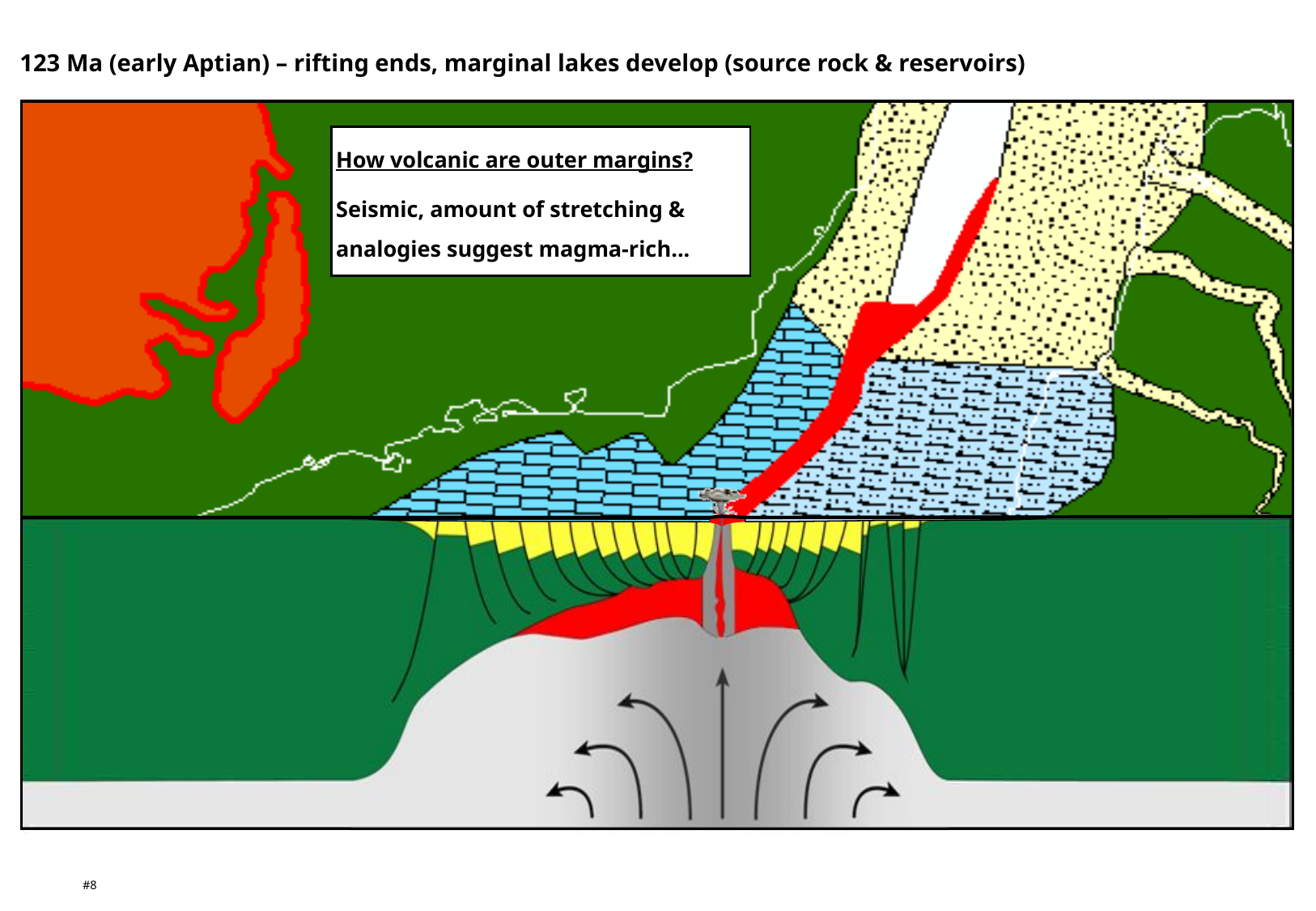

123 Ma (early Aptian) – rifting ends, marginal lakes develop (source rock & reservoirs)
How volcanic are outer margins?
Seismic, amount of stretching & analogies suggest magma-rich...
#<number>

## Slide 9
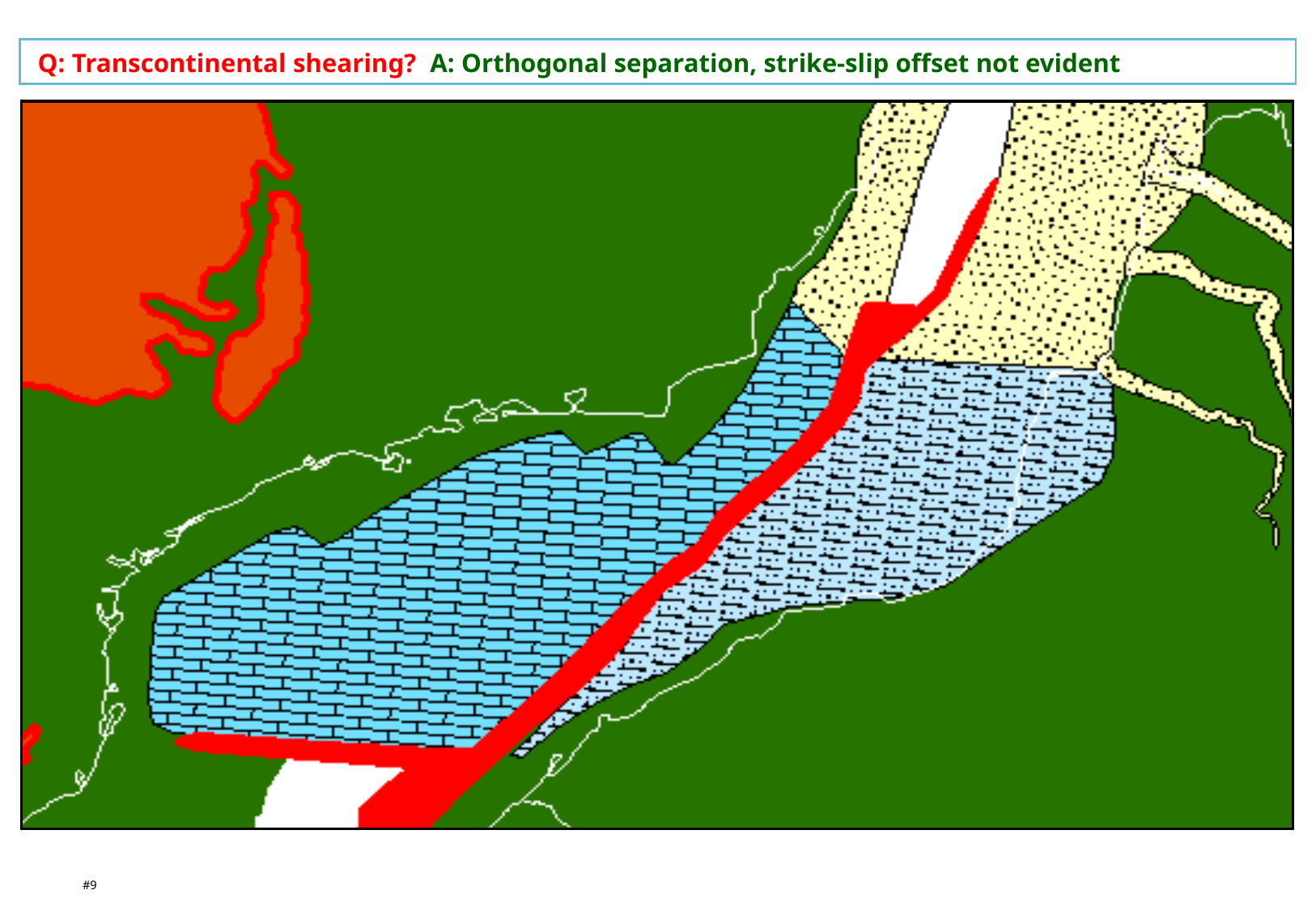

Q: Transcontinental shearing? A: Orthogonal separation, strike-slip offset not evident
123 Ma (early Aptian) – rifting ends, marginal lakes develop
#<number>

## Slide 10
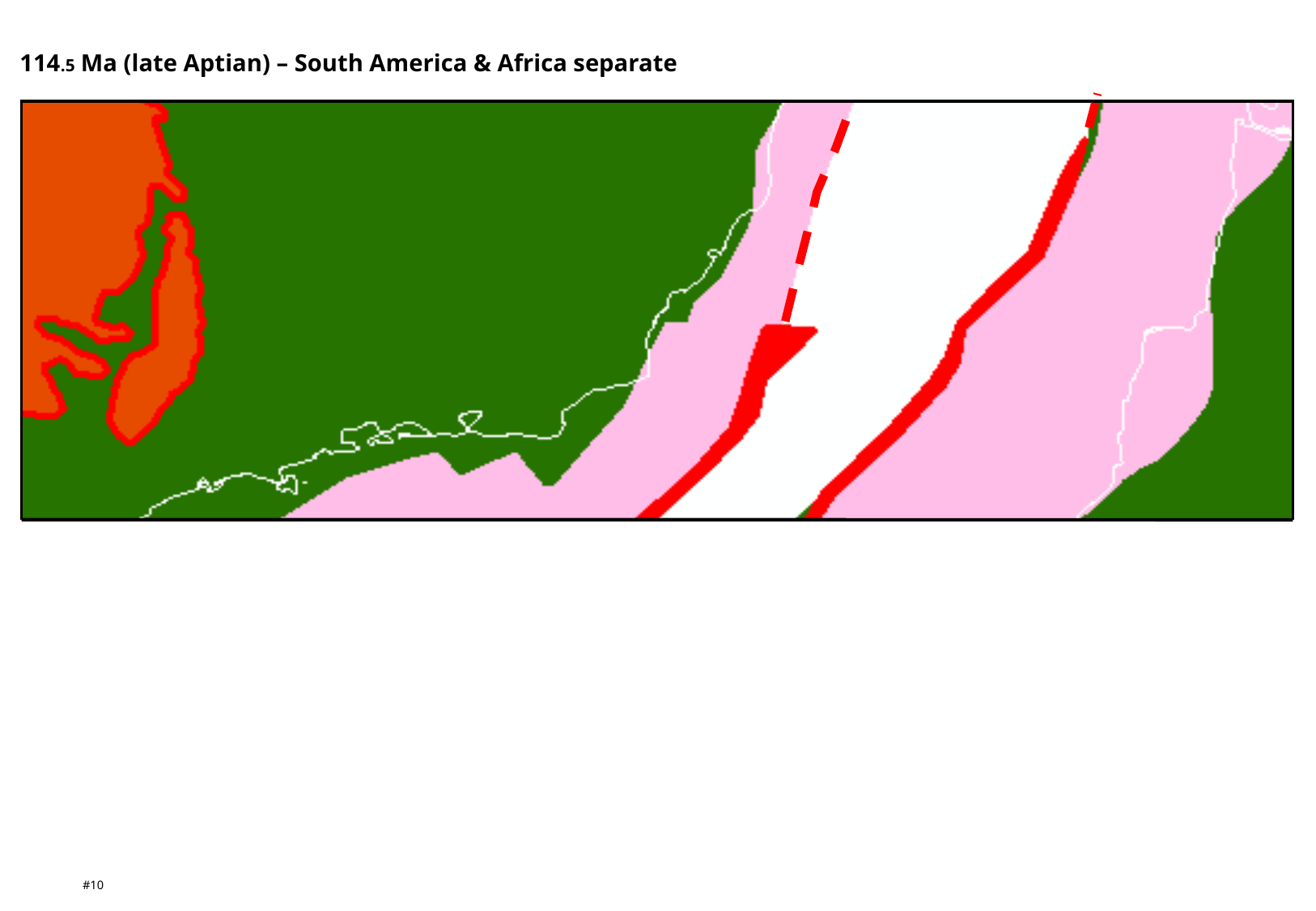

114.5 Ma (late Aptian) – South America & Africa separate
#<number>

## Slide 11
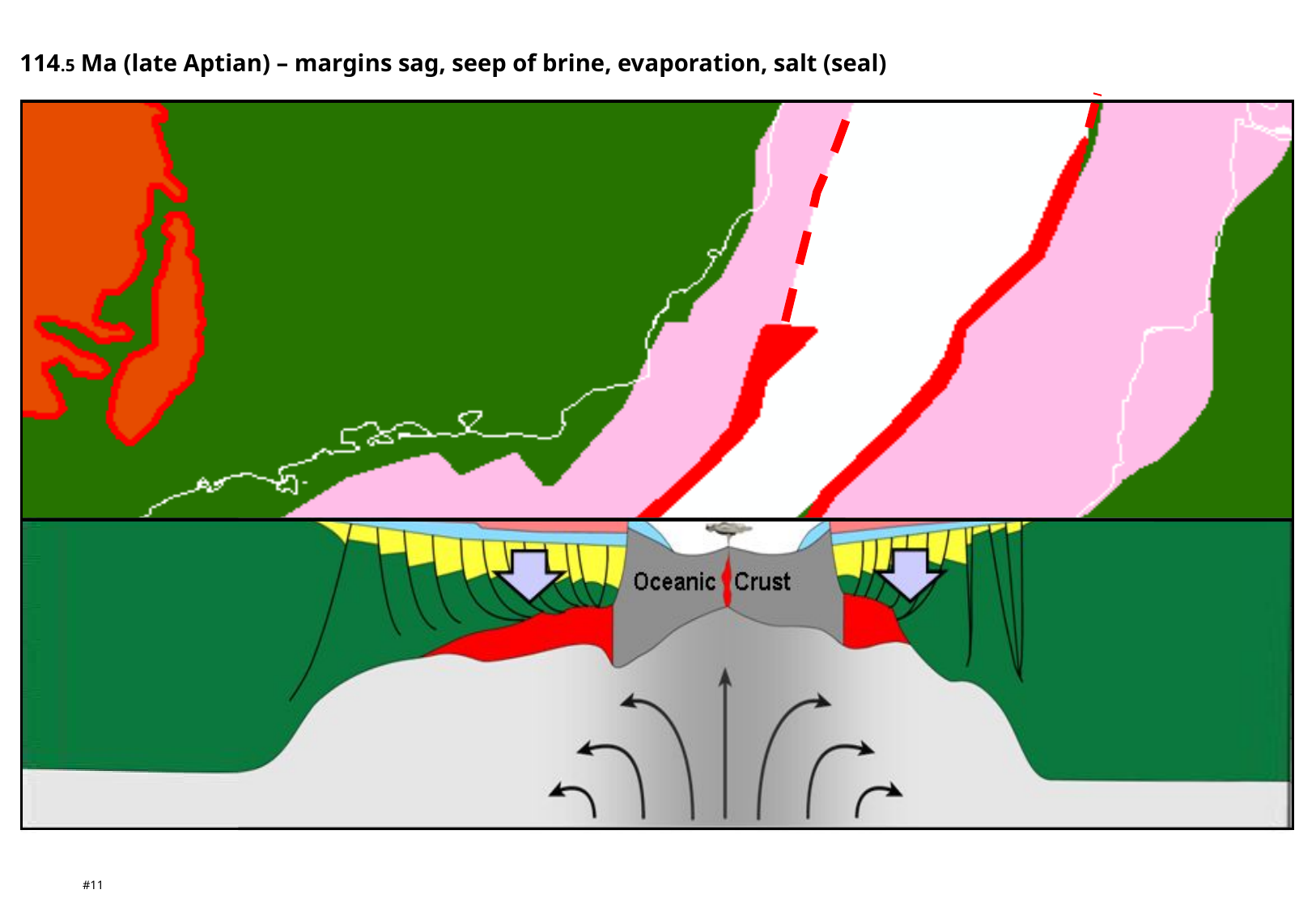

114.5 Ma (late Aptian) – margins sag, seep of brine, evaporation, salt (seal)
#<number>

## Slide 12
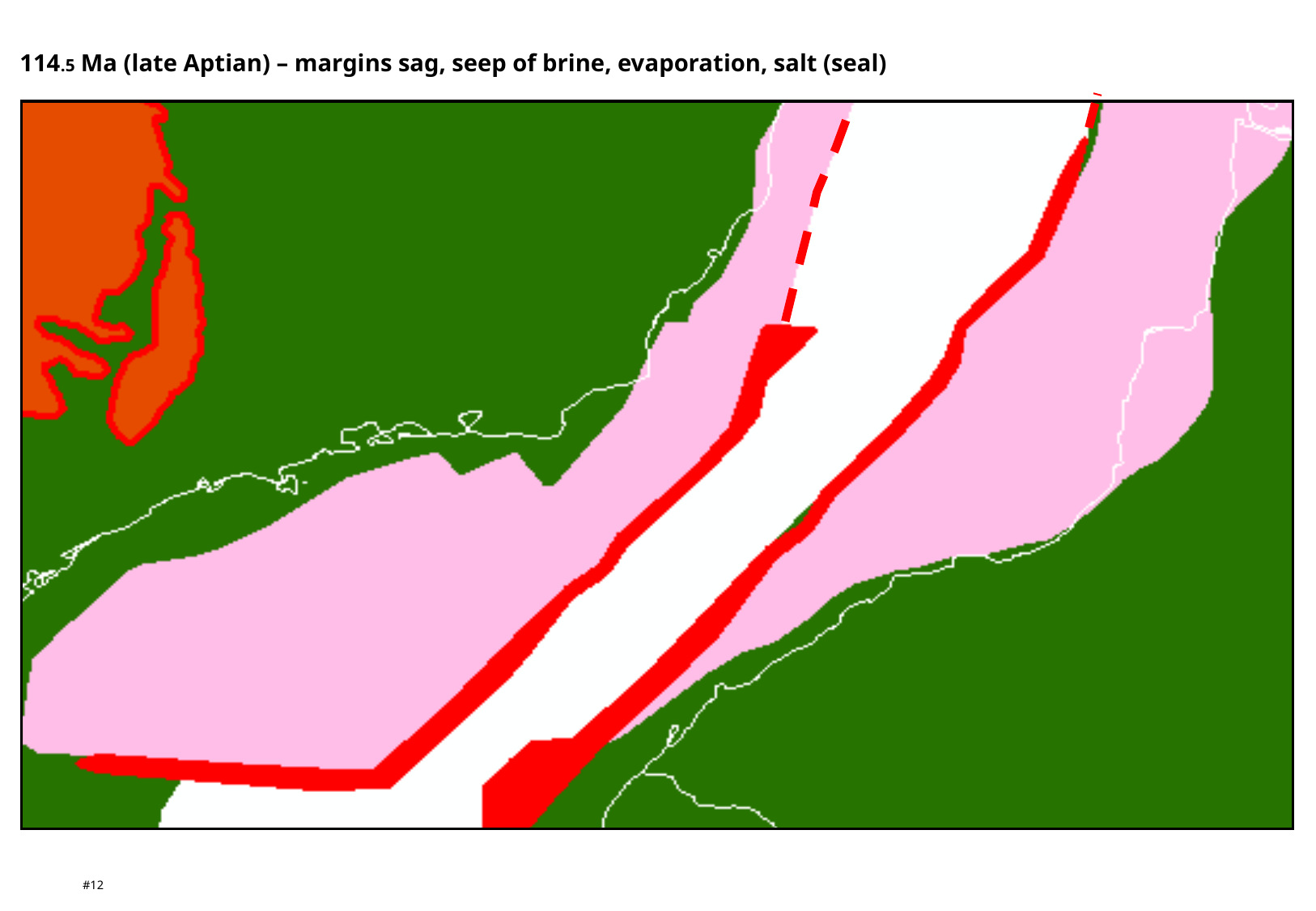

114.5 Ma (late Aptian) – margins sag, seep of brine, evaporation, salt (seal)
#<number>

## Slide 13
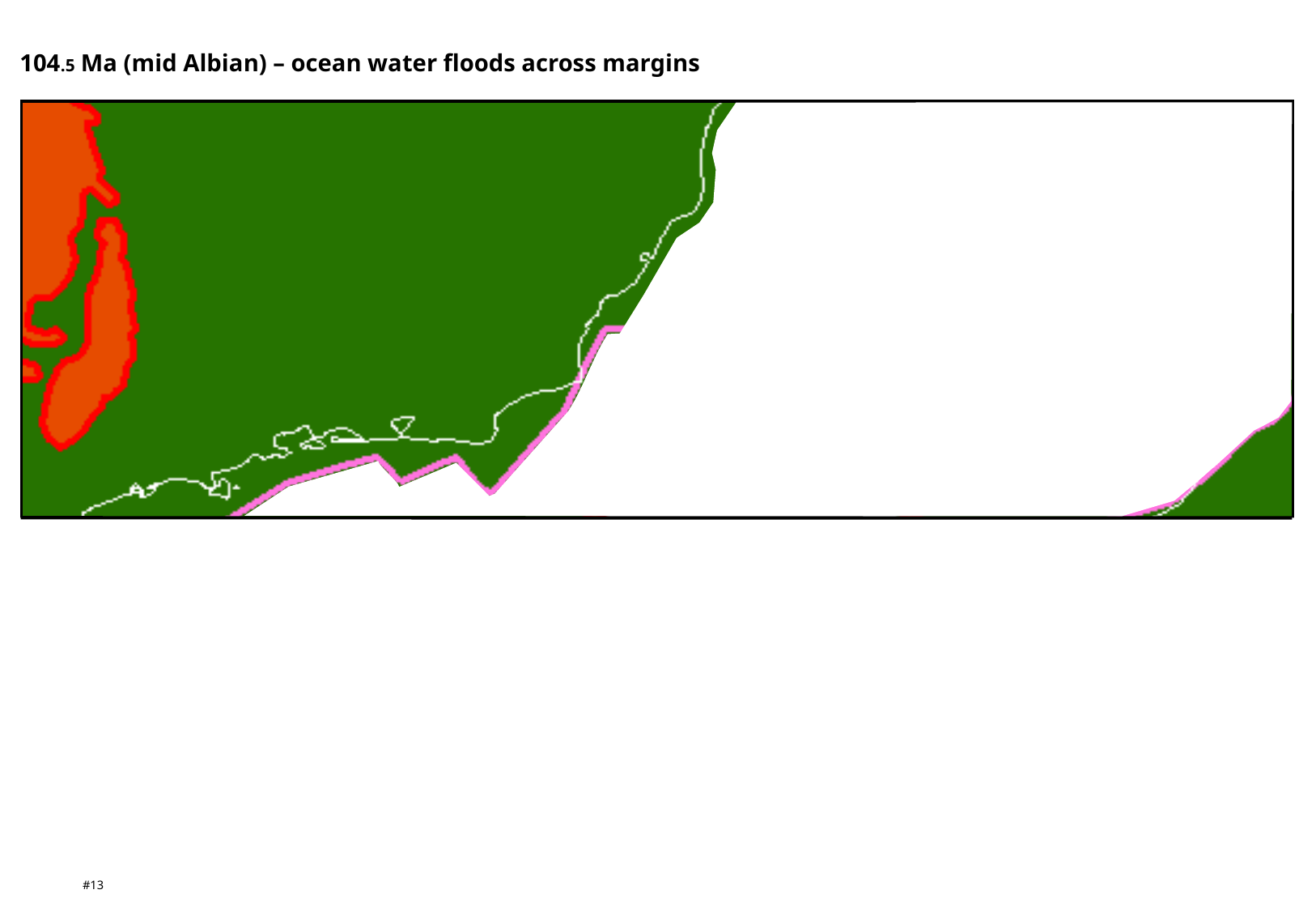

104.5 Ma (mid Albian) – ocean water floods across margins
#<number>

## Slide 14
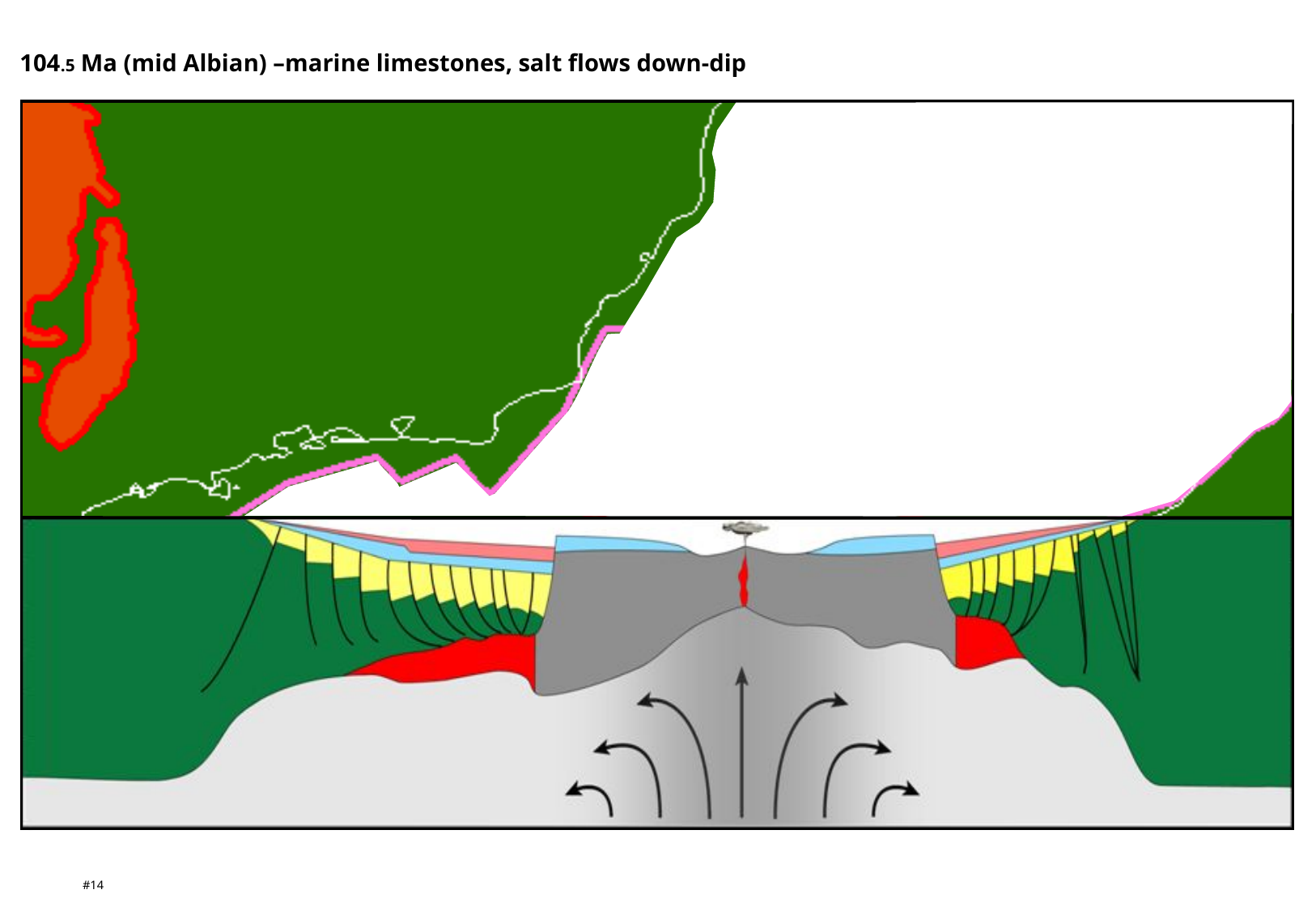

104.5 Ma (mid Albian) –marine limestones, salt flows down-dip
#<number>

## Slide 15
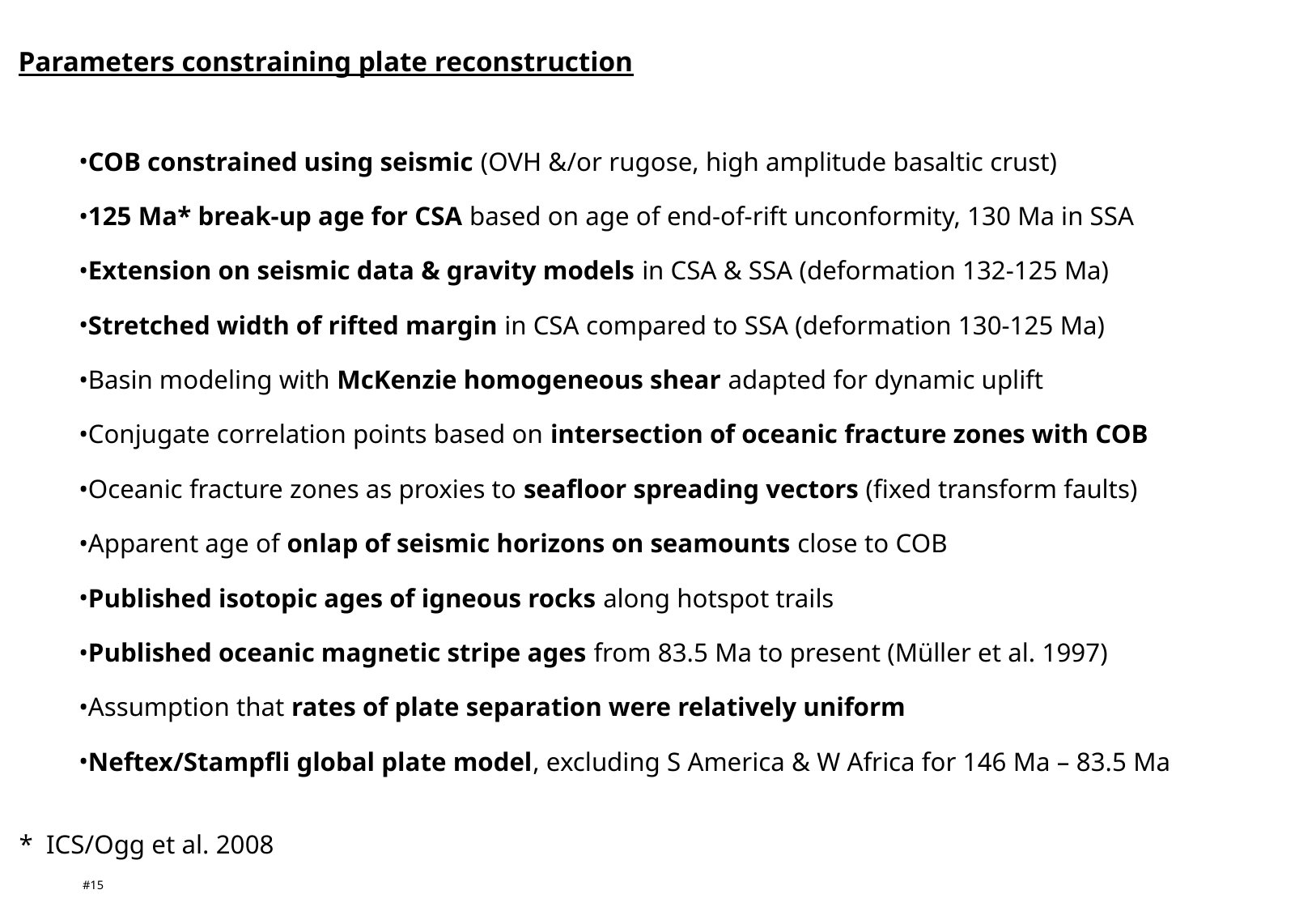

Parameters constraining plate reconstruction
COB constrained using seismic (OVH &/or rugose, high amplitude basaltic crust)
125 Ma* break-up age for CSA based on age of end-of-rift unconformity, 130 Ma in SSA
Extension on seismic data & gravity models in CSA & SSA (deformation 132-125 Ma)
Stretched width of rifted margin in CSA compared to SSA (deformation 130-125 Ma)
Basin modeling with McKenzie homogeneous shear adapted for dynamic uplift
Conjugate correlation points based on intersection of oceanic fracture zones with COB
Oceanic fracture zones as proxies to seafloor spreading vectors (fixed transform faults)
Apparent age of onlap of seismic horizons on seamounts close to COB
Published isotopic ages of igneous rocks along hotspot trails
Published oceanic magnetic stripe ages from 83.5 Ma to present (Müller et al. 1997)
Assumption that rates of plate separation were relatively uniform
Neftex/Stampfli global plate model, excluding S America & W Africa for 146 Ma – 83.5 Ma
* ICS/Ogg et al. 2008
#<number>

## Slide 16
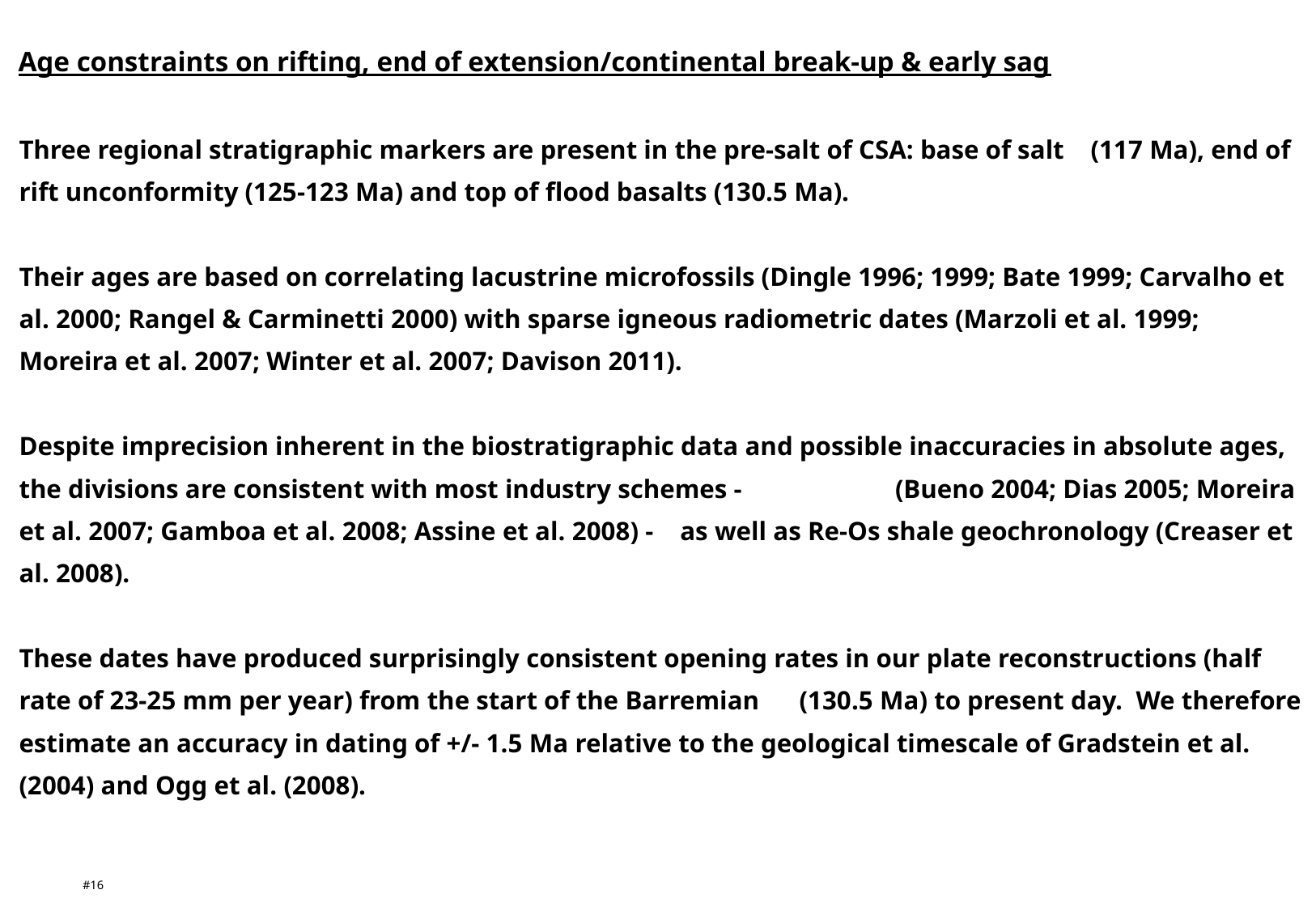

Age constraints on rifting, end of extension/continental break-up & early sag
Three regional stratigraphic markers are present in the pre-salt of CSA: base of salt (117 Ma), end of rift unconformity (125-123 Ma) and top of flood basalts (130.5 Ma).
Their ages are based on correlating lacustrine microfossils (Dingle 1996; 1999; Bate 1999; Carvalho et al. 2000; Rangel & Carminetti 2000) with sparse igneous radiometric dates (Marzoli et al. 1999; Moreira et al. 2007; Winter et al. 2007; Davison 2011).
Despite imprecision inherent in the biostratigraphic data and possible inaccuracies in absolute ages, the divisions are consistent with most industry schemes - (Bueno 2004; Dias 2005; Moreira et al. 2007; Gamboa et al. 2008; Assine et al. 2008) - as well as Re-Os shale geochronology (Creaser et al. 2008).
These dates have produced surprisingly consistent opening rates in our plate reconstructions (half rate of 23-25 mm per year) from the start of the Barremian (130.5 Ma) to present day. We therefore estimate an accuracy in dating of +/- 1.5 Ma relative to the geological timescale of Gradstein et al. (2004) and Ogg et al. (2008).
#<number>
